# Pyrrole-Containing Alkaloids from a Marine-Derived Actinobacterium *Streptomyces zhaozhouensis* and Their Antimicrobial and Cytotoxic Activities

**DOI:** 10.3390/md21030167

**Published:** 2023-03-06

**Authors:** Chang-Su Heo, Jong Soon Kang, Joo-Hee Kwon, Cao Van Anh, Hee Jae Shin

**Affiliations:** 1 Marine Natural Products Chemistry Laboratory, Korea Institute of Ocean Science and Technology, 385 Haeyang-ro, Yeongdo-gu, Busan 49111, Republic of Korea; 2 Department of Marine Biotechnology, University of Science and Technology (UST), 217 Gajungro, Yuseong-gu, Daejeon 34113, Republic of Korea; 3 Laboratory Animal Resource Center, Korea Research Institute of Bioscience and Biotechnology, 30 Yeongudanjiro, Cheongju 28116, Republic of Korea

**Keywords:** marine-derived actinomycetes, *Streptomyces zhaozhouensis*, streptopyrrole, antimicrobial, cytotoxicity, alkaloid

## Abstract

Two new alkaloids, streptopyrroles B and C (**1** and **2**), were discovered through a chemical investigation of the ethyl acetate (EtOAc) extract from a marine-derived actinomycete, *Streptomyces zhaozhouensis*, along with four known analogs (**3**–**6**). The structures of the new compounds were elucidated by spectroscopic analysis (HR-ESIMS, 1D, and 2D NMR) and a comparison of their experimental data with literature values. The new compounds were evaluated for their antimicrobial activity by standard broth dilution assay, and the tested compounds showed significant activity against Gram-positive bacteria with minimum inhibitory concentration (MIC) values ranging from 0.7 to 2.9 µM, and kanamycin was used as a positive control with MIC values ranging from <0.5 to 4.1 µM. Additionally, **1**, **3**, and **5** were evaluated for their cytotoxicity against six tumor cell lines by sulforhodamine B (SRB) assay, and these compounds displayed cytotoxic activities against all the tested cell lines, with concentration causing 50% cell growth inhibition (GI_50_) values ranging from 4.9 to 10.8 µM, while a positive control, adriamycin, showed GI_50_ values of 0.13–0.17 µM.

## 1. Introduction

Infectious diseases have been emerging as one of the significant issues endangering human health lately [1]. Although the discovery of antibiotics has alleviated the threat of pathogenic microorganisms, the rapid emergence and growth of antibiotic-resistant microbes are occurring worldwide and are rendering existing antibiotics ineffective [2,3]. According to reports, it is estimated that more than one million deaths were related to antibiotic-resistant bacteria in 2019 [4,5]. However, after peaking during the 1960s, the discovery of novel antibiotics has declined gradually, and there is an ongoing demand for the development of new antimicrobial agents [6].

The genus *Streptomyces* is renowned as the most significant contributor of leading structures for new antibiotic discovery [7]. A large number of chemical scaffolds were isolated from this genus, and many of them, such as macrolides, aminoglycosides, and tetracyclines, were approved as essential antibiotics [8,9,10]. In addition to the production of antibiotics, *Streptomyces* of various species are also able to produce cytotoxic secondary metabolites, and some currently used anti-cancer drugs are derived from this genus, for example, doxorubicin, mitomycin, and bleomycin [11]. While terrestrial microorganisms have been extensively studied, marine-derived *Streptomyces* spp. are considered an alternative source of novel bioactive compounds [12]. Under the ocean’s conditions, including high pressure, high salinity, and nutritional deprivation, marine-derived *Streptomyces* spp. undergo genetic evolution with the passage of time and yield unique secondary metabolites distinct from those of terrestrial microorganisms [13]. Over the last few years, more than 100 novel secondary metabolites have been reported from marine *Streptomyces* spp., and a significant proportion of the identified compounds have shown dual antimicrobial and cytotoxic activities [14].

Alkaloids make up one of the most significant categories of natural products isolated from marine environments [15]. Among marine alkaloids, pyrroles are a large group of interesting natural products which occur in marine environments, ranging from microorganisms to algae, sponges, and animals [16,17,18]. Pyrroles possess a flat and electron-rich aromatic ring, and their structure is susceptible to electrophilic attack and can also interact with a variety of biomolecules via hydrogen bonds as well as π–π stacking interactions [19]. Because of their intriguing structures and wide range of biomedical properties, various pyrrole-based compounds have been studied for pharmacological activities [16,20,21,22]. Streptopyrroles are one of the examples of pyrrole-containing compounds with potent antimicrobial properties and inhibitory activity on the nitrogen regulator II (NRII) histidine kinase from *Escherichia coli* [23]. This class of compounds is commonly isolated from the genus *Streptomyces* and many of them are halogenated [24].

**Figure 1:**
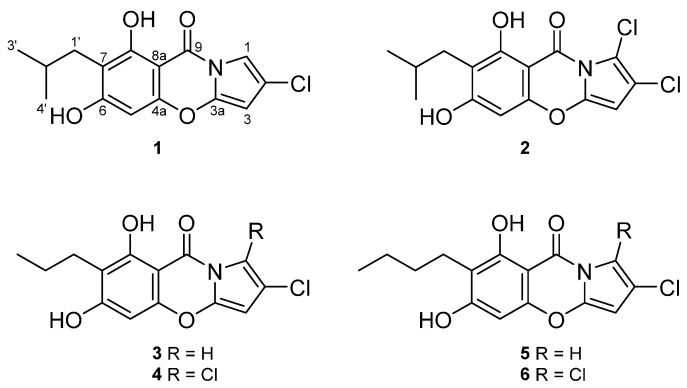
Structures of **1**–**6** isolated from *Streptomyces zhaozhouensis* 208DD-064.

In the course of our ongoing research on secondary metabolites from marine microorganisms, we isolated an actinobacterium from a sediment sample collected off Dokdo, South Korea. The strain was identified as *Streptomyces zhaozhouensis* 208DD-064 by 16S gene sequence analysis. The crude extract from the culture broth of the strain showed anti-Gram-positive bacterial and cytotoxic activities. Therefore, a mass culture was conducted to investigate novel bioactive secondary metabolites. As a result, six streptopyrroles, including two new and four known derivatives, were isolated from this strain (Figure 1). In this report, we present the extraction and structure determination, as well as the evaluation of the antimicrobial and cytotoxic activities of these compounds.

## 2. Results and Discussion

### 2.1. Structure Elucidation

Compound **1** was isolated as a white powder. The HRESIMS peaks at *m/z* 306.0534 and 308.0504 ([M-H]^−^, calculated for C_15_H_13_ClNO_4_ 306.0533) with a ratio of 3:1 determined the molecular formula of **1** as C_15_H_14_ClNO_4_, requiring 9 degrees of unsaturation and containing one chlorine atom. The IR spectrum showed an aromatic absorption band at 1536 cm^−1^, and the presence of the carbonyl group was confirmed with an absorption band at 1647 cm^−1^. The UV spectrum indicated absorption bands at 242 and 295 nm, suggesting the presence of a benzene group. The ^1^H NMR spectrum of **1** displayed signals of an aromatic singlet proton at *δ*_H_ 6.29 (s, H-5); two meta-coupling pyrrole protons at *δ*_H_ 5.87 (d, *J* = 2.1, H-3) and 7.18 (d, *J* = 2.1, H-1); a methine proton at *δ*_H_ 1.97 (m, H-2′); a methylene signal at *δ*_H_ 2.50 (d, *J* = 7.3, 2H, H_2_-1′); and two methyl groups at *δ*_H_ 0.91 (d, *J* = 6.7, 6H, H_3_-3′ and H_3_-4′) (Table 1). The ^13^C NMR and HSQC spectra of **1** exhibited the presence of a carbonyl group at *δ*_C_ 159.8 (C-9); seven non-protonated carbons at *δ*_C_ 93.2 (C-8a), 112.7 (C-7), 119.3 (C-2), 142.5 (C-3a), 154.9 (C-4a), 161.3 (C-8), and 165.8 (C-6); three aromatic protonated carbons at *δ*_C_ 90.9 (C-3), 94.2 (C-5), and 105.0 (C-1); a methine at *δ*_C_ 29.1 (C-2′); a methylene at *δ*_C_ 32.0 (C-1′); and two methyl groups at *δ*_C_ 22.9 (2C, C-3′ and C-4′) (Table 1).

**Figure 2:**
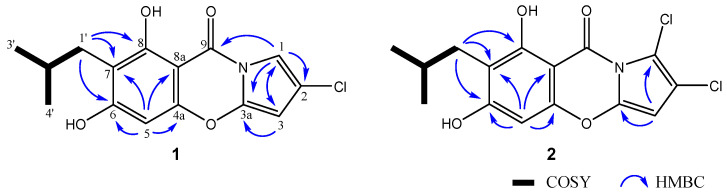
Key ^1^H–^1^H COSY and HMBC correlations for streptopyrroles (**1**) and (**2**).

The presence of an isobutyl group was determined by the ^1^H–^1^H COSY correlations from H-2′ to H_2_-1′, H_3_-3′, and H_3_-4′. The HMBC correlations from H_2_-1′ to C-6, C-7, and C-8, as well as from H-5 to C-4a, C-6, C-7, and C-8a, indicated the presence of an aromatic ring, as well as the connection of the ring to the isobutyl group at C-7. The mass and ^1^H NMR data of **1** were almost identical to those of **5** which was reported in a previous study [23]; the only difference was that the normal butyl group in **5** was changed to an isobutyl in **1**. By comparison of the chemical shifts of C-4a (*δ*_C_ 154.9), C-6 (*δ*_C_ 165.8), and C-8 (*δ*_C_ 161.3) with those reported in the literature, a moiety of the phenolic ring was established [23]. The HMBC correlations from H-1 to C-2, C-3, and C-3a, as well as from H-3 to C-1 and C-3a, identified a moiety of a five-membered pyrrole ring and a downshifted carbon at C-2 (*δ*_C_ 119.3), which suggested the presence of a chlorine atom (Figure 2). The HMBC correlation from H-1 to C-9 (*δ*_C_ 159.8) confirmed a connection of the pyrrole ring with a carbonyl group. A carboxyl, ten sp^2^ carbons, an aromatic, and a pyrrole accounted for eight out of nine indices of hydrogen deficiency, indicating the formation of an additional ring. By the detailed interpretation of the 2D NMR data of **1**, a moiety of 1,3-oxazine was interpreted by assembling the substructure from C-3a to C-4a and connecting C-8a to C-9. Therefore, the structure of **1** was elucidated as 2-chloro-6,8-dihydroxy-7-isobutyl-9*H*-pyrrolo [2,1-b] [1,3] benzoxazine-9-one and named streptopyrrole B.

Compound **2** was isolated as a white powder. Its molecular formula of C_15_H_13_Cl_2_NO_4_ was established by HRESIMS (*m/z* 340.0142 [M-H]^−^, calculated for C_15_H_12_Cl_2_NO_4_ 340.0143), suggesting nine indices of hydrogen deficiency and the substitution of a proton with a chlorine atom compare to **1**. The ^1^H NMR spectrum of **2** exhibited signals of an aromatic singlet proton at *δ*_H_ 6.25 (s, H-5); a pyrrole proton at *δ*_H_ 5.95 (s, H-3); a methine proton at *δ*_H_ 1.97 (m, H-2′); a methylene signal at *δ*_H_ 2.50 (d, *J* = 7.3, 2H, H_2_-1′); and two methyl groups at *δ*_H_ 0.91 (d, *J* = 6.7, 6H, H_3_-3′ and H_3_-4′). The ^13^C NMR in combination with the HSQC spectrum of **2** exhibited the presence of a carbonyl group at *δ*_C_ 160.9 (C-9); eight non-protonated carbons at *δ*_C_ 93.5 (C-8a), 105.1 (C-1); 113.0 (C-7), 117.3 (C-2), 141.7 (C-3a), 154.5 (C-4a), 161.6 (C-8), and 166.3 (C-6); two aromatic protonated carbons at *δ*_C_ 90.3 (C-3) and 94.1 (C-5); a methine at *δ*_C_ 29.1 (C-2′); a methylene at *δ*_C_ 32.0 (C-1′); and two methyl carbons at *δ*_C_ 22.9 (2 C, C-3′ and C-4′) (Table 1). The ^1^H and ^13^C NMR data of **2** were similar to those of **1**, except for the absence of a proton signal at the C-1 position. H-3 of **2** existed in a singlet, while the meta-coupling signal for H-l was missing. Comparing the HMBC correlations of **1** and **2**, the absence of HMBC correlations from H-1 to C-2, C-3, C-3a, and C-9 in **2** indicated that **2** is a di-chlorine-substituted analog of **1** at the C-1 position (Figure 2). Based on detailed analysis of 2D NMR data, the structure of **2** was determined as 1,2-dichloro-6,8-dihydroxy-7-isobutyl-9*H*-pyrrolo [2,1-b] [1,3] benzoxazine-9-one and named streptopyrrole C.

The structures of the known compounds (**3**–**6**) were determined as 2-chloro-6,8-dihydroxy-7-propyl-9*H*-pyrrolo [2,1-b] [1,3] benzoxazine-9-one (**3**), 1,2-dichloro-6,8-dihydroxy-7-propyl-9*H*-pyrrolo [2,1-b] [1,3] benzoxazine-9-one (**4**), 7-butyl-2-chloro-6,8-dihydroxy-9*H*-pyrrolo [2,1-b] [1,3] benzoxazine-9-one (**5**), and 7-butyl-1,2-dichloro-6,8-dihydroxy-9*H*-pyrrolo [2,1-b] [1,3] benzoxazine-9-one (**6**) by comparison of their spectroscopic data with those reported in the literature [23,25].

**Scheme 1:**
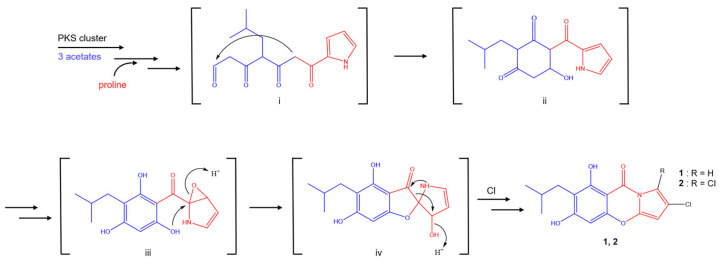
Plausible biosynthesis pathway of **1** and **2**.

In a previous study, it was reported that streptopyrrole and armeniaspirol had similar biosynthetic relationships, and their biosynthetic pathways were proposed [26,27,28]. Based on the previous reports, we present a plausible biosynthetic pathway for **1** and **2** (Scheme 1).

The polyketide synthase (PKS) clusters result in the formation of tri-ketide intermediate **i** as a starting compound. Then, the cyclization of **i** is changed to **ii**, and the following formation of the phenolic hydroxy group, as well as the epoxidation, leads to the intermediate **iii**. Afterward, the rearrangement of **iii** can form the spiro-intermediate **iv**, followed by rearomatization, as well as halogenation, and be converted into **1** and **2**.

### 2.2. Bioactivities

Since previous studies revealed that streptopyrrole derivatives display potent antibacterial activities [23], **1**–**6** were examined for their antimicrobial properties against three Gram-positive bacteria, *Bacillus subtilis* (KCTC 1021), *Micrococcus luteus* (KCTC 1915), and *Staphylococcus aureus* (KCTC 1927), and three Gram-negative bacteria, *Escherichia coli* (KCTC 2441), *Salmonella typhimurium* (KCTC 2515), and *Klebsiella pneumonia* (KCTC 2690). As a result, the new compounds **1** and **2** potently inhibited the growth of Gram-positive bacteria with MIC values ranging from 0.7 to 2.9 µM, and **3**–**6** displayed MIC values against Gram-positive bacteria ranging from 0.7 to 24.5 µM (Table 2). Especially, **1** and **3** showed stronger antimicrobial activity against *B. subtilis* and *M. luteus* than the positive control, kanamycin. However, the compounds under evaluation did not inhibit the growth of Gram-negative bacteria at a concentration of 128.0 µg/mL. This and previous studies revealed that streptopyrroles are inactive against Gram-negative bacteria [23]. In another study, antibacterial activities were investigated for armeniaspirols, which are structurally related to streptopyrrole [29]. Armeniaspirols displayed strong activities against Gram-positive bacteria in vitro, and, although adverse cardiac side effects were noted, armeniaspirol A reduced the mortality rate in mice infected with MRSA (methicillin-resistant *Staphylococcus aureus*) in vivo. These results suggested that streptopyrroles and their congeners may serve as lead structures for new antibiotics.

Compounds **1**, **3,** and **5** were evaluated for their cytotoxicity against six cancer cell lines: PC-3 (prostate), NCI-H23 (lung), HCT-15 (colon), NUGC-3 (stomach), ACHN (renal), and MDA-MB-231 (breast), which are the most common cancer types in Korea. The tested compounds displayed moderate cytotoxic activity against all the tested cell lines, with GI_50_ values ranging from 4.9 to 10.8 µM (Table 3). In a previous study, **3** was tested for its cytotoxicity against several cancer cell lines, and the compound showed moderate activity against a different group of cancer cell lines [23]. Our findings and previous report demonstrated that streptopyrroles exhibit dual antimicrobial and cytotoxic activities.

**Figure 3:**
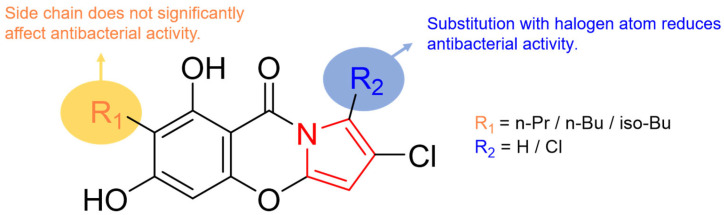
Summarization of structure–activity relationship for streptopyrroles.

The results of this study were consistent with previous studies [23], indicating that monochloride-substituted streptopyrroles display overall stronger antibacterial activity than dichloride-substituted streptopyrroles. This result could be explained by the fact that pyrrole is an electron-rich aromatic system, and the presence of electron-withdrawing groups (halogen atoms) might reduce their bioactivity (Figure 3). The length and branching in the side chain did not significantly affect antibacterial activity (**1**, **3**, **5**). Due to the limited amount of samples (**2**, **4**, **6**), it was not possible to check the cytotoxicity of all the isolated compounds, and the structure–activity relationship of the cytotoxicity needs to be thoroughly studied. Therefore, further studies are necessary to understand the effect of electron-withdrawing groups on streptopyrrole analogs. 

## 3. Materials and Methods

### 3.1. General Experimental Procedures

The 1D and 2D NMR spectra were obtained using a Bruker 600 MHz spectrometer (Bruker BioSpin GmbH, Rheinstetten, Germany). UV–Vis spectra were acquired by a Shimadzu UV-1650PC spectrophotometer (Shimadzu Corporation, Kyoto, Japan). IR spectra were measured using a JASCO FT/IR-4100 spectrophotometer (JASCO Corporation, Tokyo, Japan). High-resolution ESIMS experiments were carried out using a hybrid ion trap time-of-flight mass spectrometer (Shimadzu LC/MS-IT-TOF, Kyoto, Japan). HPLC was conducted using an UltiMate 3000 (Thermo Scientific, Waltham, MA, USA). Semi-preparative HPLC was performed on an ODS column (YMC-Pack-ODS-A, 250 × 10 mm i.d, 5 µM, Kyoto, Japan).

### 3.2. Isolation of the Microorganisms from Marine Sediment Samples

Marine sediment samples were collected offshore of Dokdo Island, Republic of Korea, during expeditions in August 2020. The sediment samples were obtained at ~200 m below the water surface by a grab sampler. After collection, the sediments were put into sterile 50 mL conical tubes and stored at 5 °C while being returned to the laboratory. *Actinomycetes* can survive in a severe environment with high temperatures because of their spore formation [30]. Therefore, selective heating pretreatment was carried out to eliminate unwanted microorganisms. A 1.0 g amount of each collected sediment sample was placed in a sterile plate and kept in an incubator at 60 °C for 30 min. After the heat treatment, 0.1 g of sediment was serially diluted to 10^−1^, 10^−2^, and 10^−3^ by sterile seawater and then each aliquot (100 µL) was spread on Bennett’s agar (BN), actinomycetes isolation agar (AIA), and humic acid-vitamin agar (HV) medium. The plates were incubated in a BOD (bio-oxygen demand) incubator at 28 °C for 7~28 days until colonies were visible. After incubation, colonies were selected and transferred onto new BN agar plates. The purification was conducted several times until single pure colonies were obtained. 

### 3.3. Isolation and Identification of the Strain 208DD-064

The strain 208DD-064 was isolated from humic acid-vitamin agar incubated for 14 days and was identified as *Streptomyces zhaozhouensis* based on morphological characteristics and 16S rRNA gene sequence analysis (GenBank accession number OQ291585).

### 3.4. Small-Scale Cultivation of the Strain 208DD-064 and the Test for Nutrient Supply Effect

Before the mass culture of the producing strain, the effect of nutrient supply was tested to check the production of bioactive compounds by culturing the strain in nutrient-rich (BN) and nutrient-poor media (modified BN). A small-scale culture of the strain 208DD-064 was carried out to confirm the production of unusual bioactive secondary metabolites. The strain 208DD-064 in agar plate was inoculated to a 100 mL flask containing 50 mL BN liquid medium (BN broth, 10 g/L glucose, 2 g/L tryptone, 1 g/L yeast extract, 1 g/L beef extract, 5 g/L glycerol, 32.0 g/L sea salt, pH 7.0 before sterilization), and the flask was incubated in shaking incubator at 28 °C for 14 days under 120 rpm. The culture medium was extracted twice with an equal volume of EtOAc (50 mL × 2) and concentrated in vacuo to yield a crude extract. In the preliminary screening, the crude extract from the small-scale culture showed anti-Gram-positive bacterial and cytotoxic activities. Another small-scale culture of the strain 208DD-064 was carried out to test the nutrient supply effect on the strain using a nutrient-poor medium (modified BN broth, 1.0 g/L glucose, 0.2 g/L tryptone, 0.1 g/L yeast extract, 0.1 g/L beef extract, 0.5 g/L glycerol, 32.0 g/L sea salt, pH 7.0 before sterilization). After extraction, the extract was analyzed by NMR spectroscopy and displayed unusual signals from ^1^H NMR data, accounting for streptopyrroles. 

### 3.5. Fermentation of the Strain 208DD-064 and Extraction and Isolation of Metabolites 

The seed and mass cultures of the strain 208DD-064 were carried out using a modified Bennett’s medium. A single colony of the strain from an agar plate was inoculated aseptically into a 2.0 L flask filled with 1.0 L of modified BN broth. After that, the strain was incubated at 28 °C for 7 days on a rotary shaker at 120 rpm and then the culture broth was transferred to a 100 L fermenter filled with 70 L of modified BN broth. The mass culture was conducted for 14 days at 28 °C and then harvested. The culture broth (70 L) was centrifuged at 60,000 rpm, and the supernatant was extracted twice with an equal volume of EtOAc (70 L × 2). The EtOAc layer was evaporated to yield a crude extract (14.0 g). The crude extract was subjected to an ODS column chromatography followed by a stepwise gradient elution with MeOH in H_2_O (1:4, 2:3, 3:2, 4:1, and 100:0, *v/v*). The fraction eluted with 100% MeOH was purified by a semi-preparative reversed-phase HPLC (YMC-Pack-ODS-A, 250 × 10 mm i.d, 5 µm, flow rate 2.0 mL/min, RI detector) using a gradient elution with acetonitrile (MeCN) in H_2_O (75% to 100% MeCN, 0.0 to 120.0 min) to yield **1** (3.0 mg, *t*_R_ = 44 min), **2** (0.7 mg, *t*_R_ = 51 min), **3** (6.1 mg, *t*_R_ = 37 min), **4** (1.3 mg, *t*_R_ = 43 min), **5** (4.6 mg, *t*_R_ = 46 min), and **6** (0.9 mg, *t*_R_ = 52 min).

Streptopyrrole B (**1**): a white powder; IR *ν*_max_ 2927, 2355, 1647, 1536, 1053; UV (MeOH) λ_max_ (log *ε*) 242 (4.53), 295 (4.20) nm; HRESIMS *m/z* 306.0534 ([M-H]^−^, (calculated for C_15_H_13_ClNO_4_ 306.0533); ^1^H and ^13^C NMR data (CD_3_OD, 600 MHz and 150 MHz, respectively), Table 1.

Streptopyrrole C (**2**): a white powder; IR *ν*_max_ 3364, 2946, 2833, 1649, 1413, 1010; UV (MeOH) λ_max_ (log *ε*) 245 (4.60), 298 (4.25), 330 (3.60) nm; HRESIMS *m/z* 340.0142 [M-H]^−^, (calculated for C_15_H_12_Cl_2_NO_4_ 340.0143); ^1^H and ^13^C NMR data (CD_3_OD, 600 MHz and 150 MHz, respectively), Table 1.

Compound **3**: a white powder; ^1^H NMR (CD_3_OD, 600 MHz) *δ*_H_ 7.11 (d, *J* = 1.7, 1H, H-1), 6.23 (s, 1H, H-5), 5.81 (d, *J* = 1.7, 1H, H-3), 2.57 (t, *J* = 7.5, 2H, H_2_-1′), 1.54 (m, 2H, H_2_-2′), 0.95 (t, *J* = 7.3, 3H, H_3_-3′); LRESIMS *m/z* 292.0 [M-H]^−^.

Compound **4**: a white powder; ^1^H NMR (CD_3_OD, 600 MHz) *δ*_H_ 6.22 (*s*, 1H, H-5), 5.94 (*s*, 1H, H-3), 2.59 (t, *J* = 7.6, 2H, H_2_-1′), 1.54 (m, 2H, H_2_-2′), 0.95 (t, *J* = 7.3, 3H, H_3_-3′); LRESIMS *m/z* 325.9 [M-H]^−^.

Compound **5**: a white powder; ^1^H NMR (CD_3_OD, 600 MHz) *δ*_H_ 7.12 (*d*, *J* = 2.0, 1H, H-1), 6.23 (s, 1H, H-5), 5.81 (d, *J* = 2.0, 1H, H-3), 2.59 (t, *J* = 7.5, 2H, H_2_-1′), 1.48 (m, 2H, H_2_-2′), 1.36 (m, 2H, H_2_-3′), 0.94 (t, *J* = 7.3, 3H, H_3_-4′); LRESIMS *m/z* 306.0 [M-H]^−^. 

Compound **6**: a white powder; ^1^H NMR (CD_3_OD, 600 MHz) *δ*_H_ 6.24 (*s*, 1H, H-5), 5.94 (*s*, 1H, H-3), 2.62 (t, *J* = 7.6, 2H, H_2_-1′), 1.49 (m, 2H, H_2_-2′), 1.38 (m, 2H, H_2_-3′), 0.94 (t, *J* = 7.3, 3H, H_3_-4′); LRESIMS *m/z* 339.9 [M-H]^−^. 

### 3.6. Antibacterial Assay

Antibacterial assay was performed in a 96-well plate (SPL Life Sciences, Pocheon, South Korea) using the broth microdilution method as described by the Clinical and Laboratory Standards Institute [31]. Gram-positive and Gram-negative bacteria were purchased from the Korean Collection for Type Cultures (KCTC, Daejeon, Korea). Briefly, the bacteria were incubated in Mueller–Hinton broth (MHB) for a day. Test compounds (**1**–**6**) were dissolved in dimethyl sulfoxide (DMSO), and a serial twofold dilution of the compounds was prepared with MHB (100 µL) in each well in the range of 0.5–256 (µg/mL). The broth containing the bacteria (100 µL) was added to the well, and the final concentration of bacteria was adjusted to 5 × 10^5^ CFU/mL by comparison with the McFarland standard. The plates were incubated for 20 h at 37 °C, and MIC was evaluated by the lowest concentration at which the bacteria did not exhibit any apparent growth.

### 3.7. Sulforhodamine B (SRB) Assay for Cytotoxicity Test

Cytotoxicity test was performed using a sulforhodamine B (SRB) assay according to the published procedures [32]. Statistical analysis was evaluated by one-way ANOVA followed by Dunnett’s *t*-test, and the GI50 values were determined by the software of GraphPad Prism 8 (GraphPad Software Inc., San Diego, CA, USA). Briefly, the human cancer cell lines were obtained from the Japanese Cancer Research Resources Bank (JCRB) (NUGC-3, JCRB Cell Bank/Cat. #JCRB0822) and American Type Culture Collection (ATCC) (PC-3, ATCC/Cat. #CRL-1435; MDA-MB-231, ATCC/Cat. #HTB-26; ACHN, ATCC/Cat. #CRL-1611; NCI-H23, ATCC/Cat. #CRL-5800; HCT-15, ATCC/Cat. #CCL-225). The cancer cell lines were cultured in RPMI 1640 supplemented with 10% fetal bovine serum (FBS) at 37 °C under a humidified atmosphere of 5% CO_2_. Then, 8,000 cells/well were seeded onto a 96-well plate, and the cells were treated with **1**, **3**, and **5**, vehicle control (0.1% DMSO), and positive control (adriamycin). After incubation for 48 h, the cultures were fixed using 50% trichloroacetic acid (50 μg/mL) and were marked with 0.4% sulforhodamine B in 1% acetic acid. Unbound dye was washed using 1% acetic acid, and protein-bound dye was collected with 10 mM Tris base (pH 10.5) to measure the optical density. Absorbance was measured at 540 nm with a VersaMax microplate reader from Molecular Devices (LLC, Sunnyvale, CA, USA).

## 4. Conclusions

The genus *Streptomyces* is renowned as a promising source of bioactive chemical scaffolds. By bioassay-guided screening, the strain *S. zhaozhouensis* 208DD-064 was selected for further study, and chemical investigation of a large-scale culture resulted in the isolation of two new and four known streptopyrrole derivatives. The structures of the new compounds were determined by detailed analysis of HR-ESIMS, 1D, and 2D NMR data. Analysis of the spectroscopic data revealed a structural feature of new streptopyrroles (**1** and **2**), including a branched side chain with a pyrrole moiety. This is the first report on streptopyrrole derivatives with a branched side chain. All the isolated compounds were tested for their antimicrobial activities. The new compounds (**1** and **2**) showed significant antimicrobial activity against Gram-positive bacteria. Additionally, **1**, **3**, and **5** were examined for their cytotoxicity against six cancer cell lines, and the tested compounds exhibited potent cytotoxicity. The results demonstrated that streptopyrroles may serve as lead structures for the development of new antibacterial and anti-cancer drugs. Considering the antimicrobial activity of streptopyrroles (**1**–**6**) against Gram-positive bacteria, it is suggested that dichloride-substituted streptopyrroles exhibit overall weaker antibacterial activity than monochloride-substituted streptopyrroles. However, further research is needed to better comprehend the structure–activity relationship of streptopyrroles.

## Figures and Tables

**Table 1:** ^1^H and ^13^C NMR data for **1** and **2** in CD_3_OD at 600 MHz for ^1^H and 150 MHz for ^13^C (*δ* in ppm, *J* in Hz).

No	1		2	
	*δ*_H_, Mult (*J* in Hz)	*δ*_C,_ Type	*δ*_H_, Mult (*J* in Hz)	*δ*_C,_ Type
1	7.18, d (2.1)	105.0, CH		105.1, C
2		119.3, C		117.3, C
3	5.87, d (2.1)	90.9, CH	5.95, s	90.3, CH
3a		142.5, C		141.7, C
4a		154.9, C		154.5, C
5	6.29, s	94.2, CH	6.25, s	94.1, CH
6		165.8, C		166.3, C
7		112.7, C		113.0, C
8		161.3, C		161.6, C
8a		93.2, C		93.5, C
9		159.8, C		160.9, C
1′	2.50, d (7.3)	32.0, CH_2_	2.50 d (7.3)	32.0, CH_2_
2′	1.97, m	29.1, CH	1.97, m	29.1, CH
3′	0.91, d (6.7)	22.9, CH_3_	0.91, d (6.7)	22.9, CH_3_
4′	0.91, d (6.7)	22.9, CH_3_	0.91, d (6.7)	22.9, CH_3_

**Table 2:** Antibacterial activity of **1**–**6.**.

Strains	MIC (µM)						
	1	2	3	4	5	6	Kanamycin
*Bacillus subtilis*	0.8	2.9	0.9	24.5	6.5	5.9	1.0
*Micrococcus luteus*	0.8	0.7	0.9	0.8	0.8	0.7	4.1
*Staphylococcus aureus*	0.8	0.7	0.9	1.5	0.8	1.5	<0.5
*Escherichia coli*	-	-	-	-	-	-	<0.5
*Salmonella typhimurium*	-	-	-	-	-	-	4.1
*Klebsiella pneumonia*	-	-	-	-	-	-	1.0

Kanamycin as a positive control. MIC values are minimum inhibitory concentrations. ‘’-’’ means not active at the concentration of 128.0 µg/mL.

**Table 3:** Growth inhibition (GI_50_, µM) of **1**, **3,** and **5** against human cancer cell lines.

Cell Lines	GI_50_ (µM)			
	1	3	5	Adriamycin
ACHN	4.9	6.0	10.5	0.17
MDA-MB-231	6.5	7.1	10.7	0.14
PC-3	6.3	7.7	10.4	0.15
NUGC-3	5.3	6.4	10.4	0.15
NCI-H23	6.1	6.8	10.8	0.13
HCT-15	6.6	7.7	10.7	0.16

Adriamycin as a positive control. GI_50_ values are the concentration corresponding to 50% growth inhibition. **2**, **4**, and **6** were not tested due to the limited amount of samples.

## Data Availability

The data presented in the article are available in the Supplementary Materials.

## Supplementary Materials

The followings are available online at https://www.mdpi.com/article/10.3390/md21030167/s1, Figure S1: Structures of streptopyrroles (**1**–**6**), Figure S2: Comparison of chemical shifts between the new compounds (**1** and **2**) and known analogs (**3** and **4**), Figures S3–16: IR, HR-ESIMS data, ^1^H NMR, ^13^C NMR, HSQC, ^1^H–^1^H COSY, HMBC NMR spectra of **1** and **2**, Figures S17–S24: MS and ^1^H NMR spectra of known analogs (**3**–**6**), Table S1: Results of the cytotoxicity test of **1**, **3**, and **5**.
