# Supplementary material for: Pyrrole-Containing Alkaloids from a Marine-Derived Actinobacterium Streptomyces zhaozhouensis and Their Antimicrobial and Cytotoxic Activities
Source: Mar Drugs. 2023 Mar 6;21(3):167. doi: 10.3390/md21030167 (PMC10054583; doi:10.3390/md21030167)
Supplement: Supplementary file 1 [file marinedrugs-21-00167-s001.zip › marinedrugs-2226281-supplementary.pdf]

Pyrrole-Containing Alkaloids from a Marine-Derived Actinobacterium *Streptomyces zhaozhouensis* and Their Antimicrobial and Cytotoxic Activities

Contents

Figure S1. Structures of **1-6** isolated from *Streptomyces zhaozhouensis* 208DD-064 2

Figure S2. Comparison of chemical shifts between the new compounds (**1**, **2**) and known analogs 3

Figure S3. IR spectrum of **1** 4

Figure S4. HRESMS data of **1** 5

Figure S5. ^{1}H NMR spectrum of **1** 6

Figure S6. ^{13}C NMR spectrum of **1** 6

Figure S7. HSQC spectrum of **1** 7

Figure S8. ^{1}H-^{1}H COSY spectrum of **1** 8

Figure S9. HMBC spectrum of **1** 9

Figure S10. IR spectrum of **2** 10

Figure S11. HRESIMS data of **2** 11

Figure S12. ^{1}H NMR spectrum of **2** 12

Figure S13. ^{13}C NMR spectrum of **2** 12

Figure S14. HSQC spectrum of **2** 13

Figure S15. ^{1}H-^{1}H COSY spectrum of **2** 14

Figure S16. HMBC spectrum of **2** 15

Figure S17. LRESIMS data of **3** 16

Figure S18. ^{1}H NMR spectrum of **3** 16

Figure S19. LRESIMS data of **4** 17

Figure S20. ^{1}H NMR spectrum of **4** 17

Figure S21. LRESIMS data of **5** 18

Figure S22. ^{1}H NMR spectrum of **5** 18

Figure S23. LRESIMS data of **6** 19

Figure S24. ^{1}H NMR spectrum of **6** 19

Table S1. Results of the cytotoxicity test of **1**,**3** and **5** 20

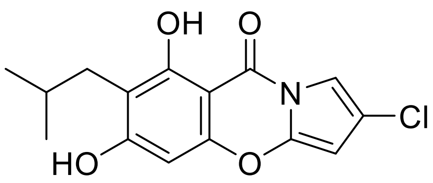

1

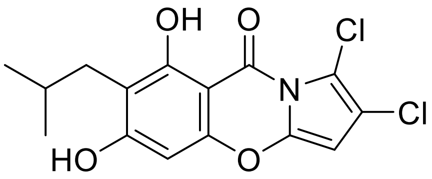

2

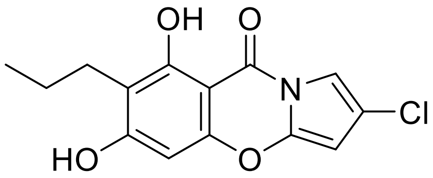

3

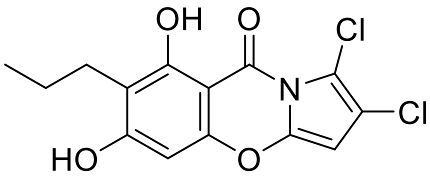

4

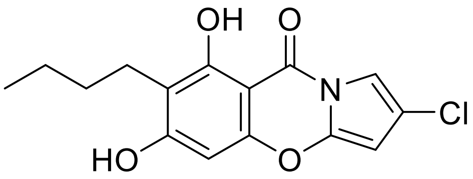

5

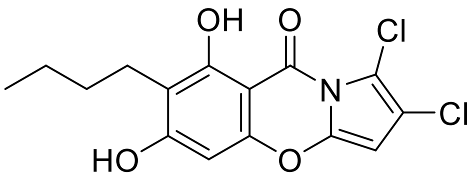

6

Figure S1. Structures of **1-6** isolated from *Streptomyces zhaozhouensis* 208DD-064.

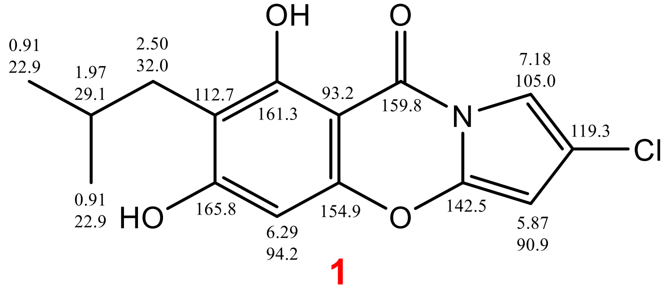

1

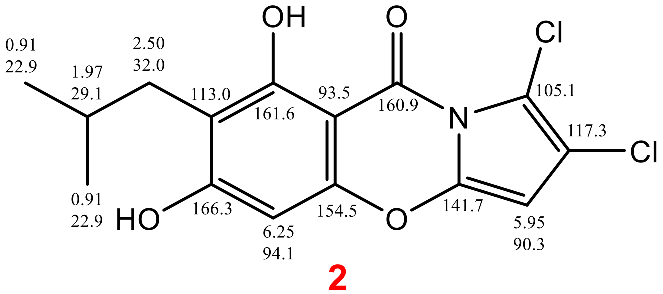

2

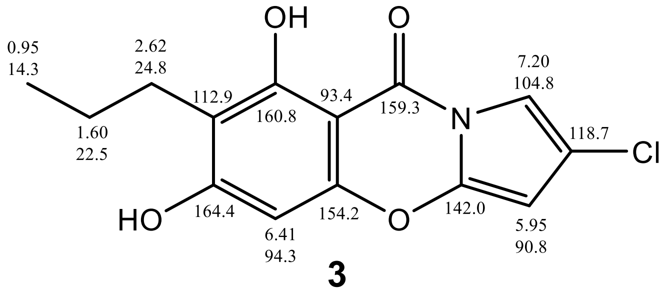

3

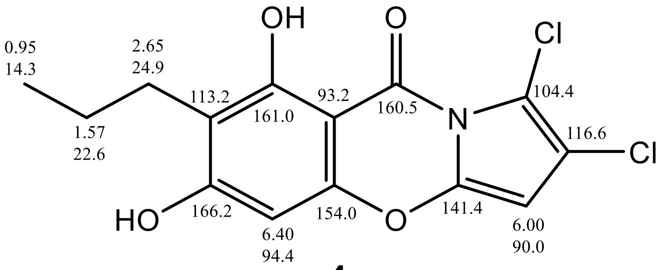

4

Figure S2. Comparison of chemical shifts between the new compounds (**1** and **2** in CD_3OD) and known analogs (**3** and **4** in acetone-d_6).

NMR data for compounds **3**, **4** were adopted from Ref. "Novel streptopyrroles from *Streptomyces rimosus* with bacterial protein histidine kinase inhibitory and antimicrobial activities". *J. Antibiot.* **2000,** *53*, 1-11.

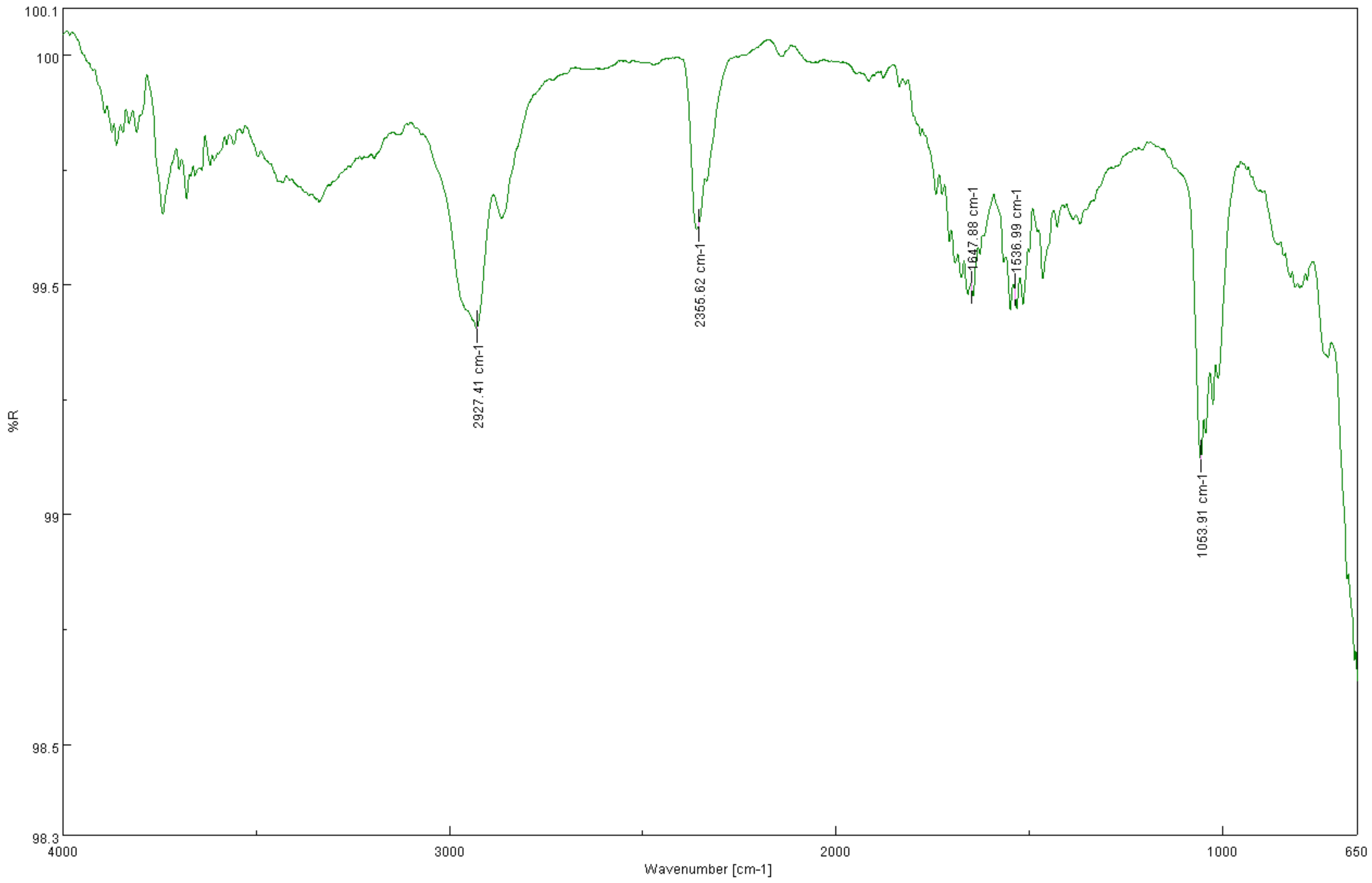

Figure S3. IR spectrum of **1**.

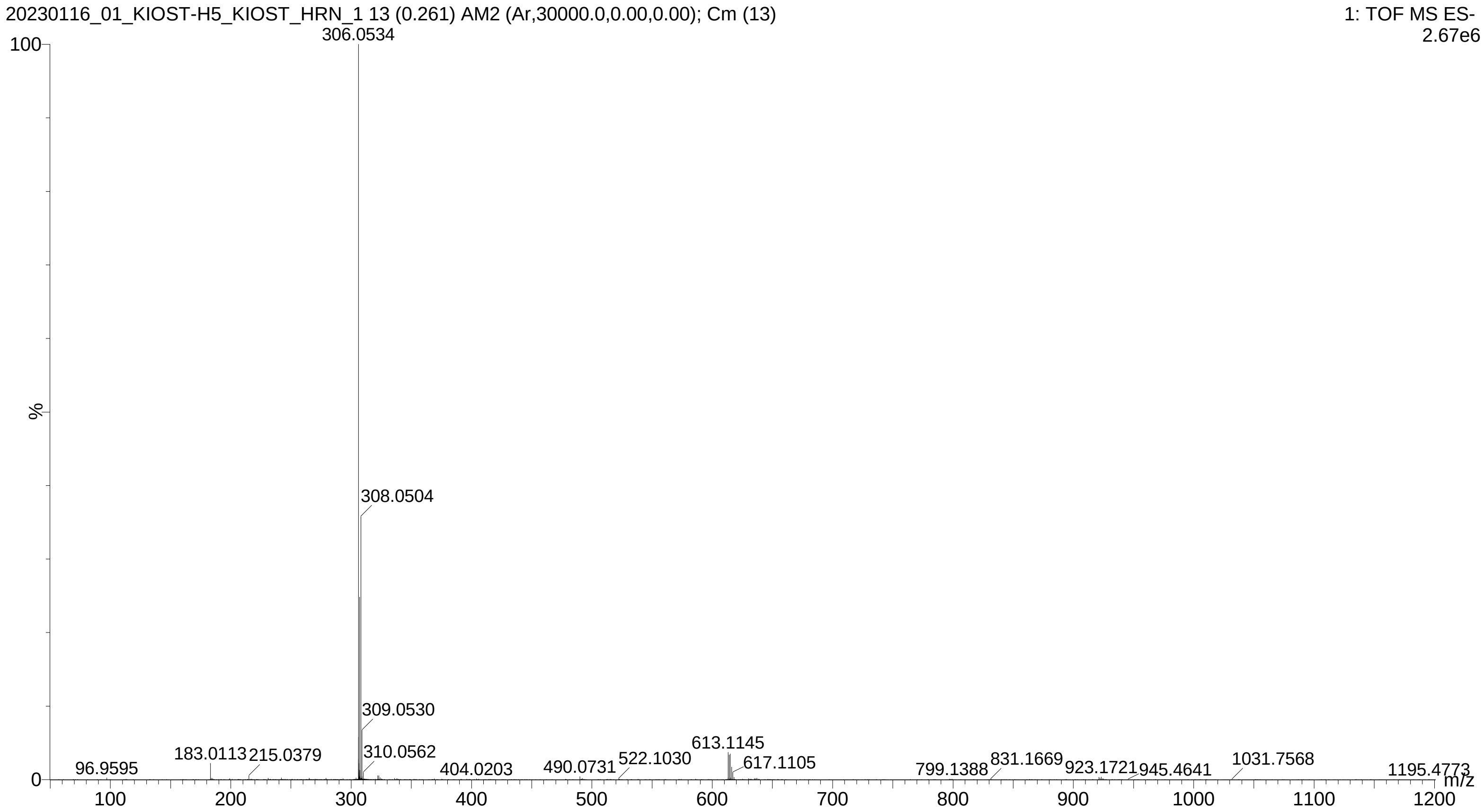

Elements Used:

C: 0-20 H: 0-180 N: 0-2 O: 0-5 Cl: 0-2

Minimum:				-9.0				
Maximum:		5.0	5.0	100.0				
Mass	Calc. Mass	mDa	PPM	DBE	i-FIT	Norm	Conf(%)	Formula
306.0534	306.0533	0.1	0.3	9.5	1496.9	n/a	n/a	C15 H13 N O4 Cl

Figure S4. HRESMS data of **1**.

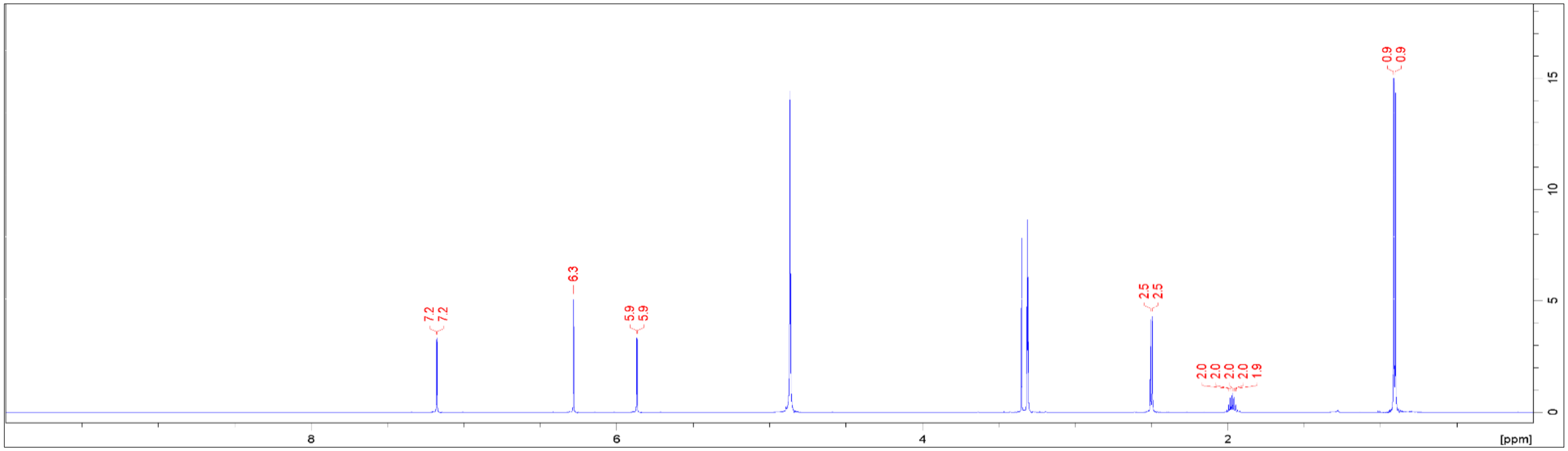

Figure S5. ^{1}H NMR spectrum of **1** (CD_3OD).

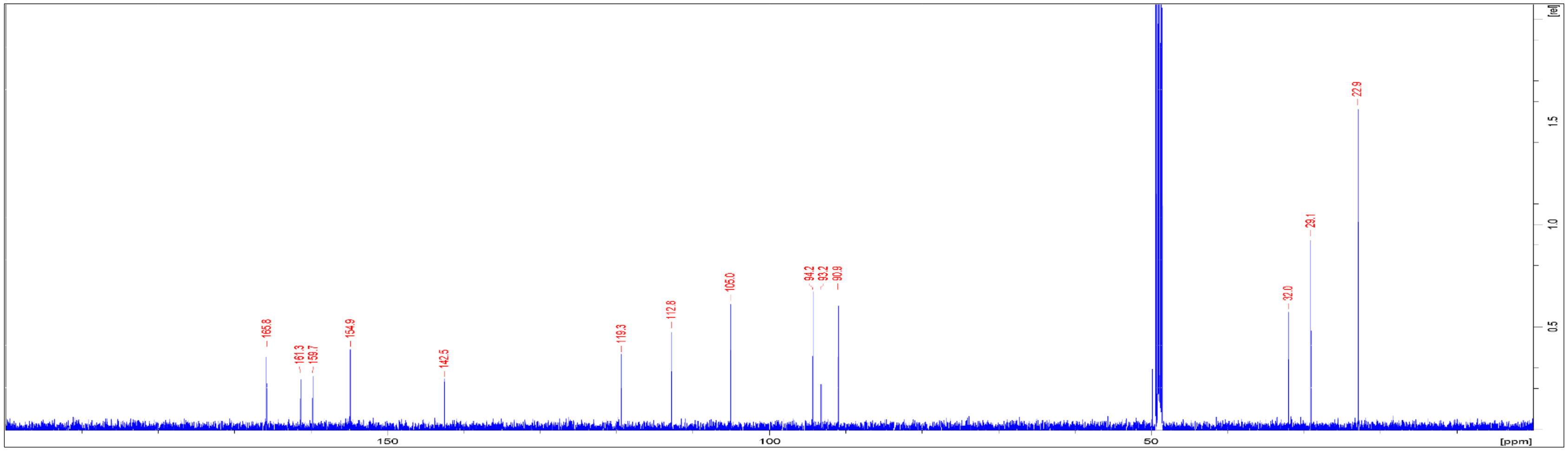

Figure S6. ^{13}C NMR spectrum of **1** (CD_3OD).

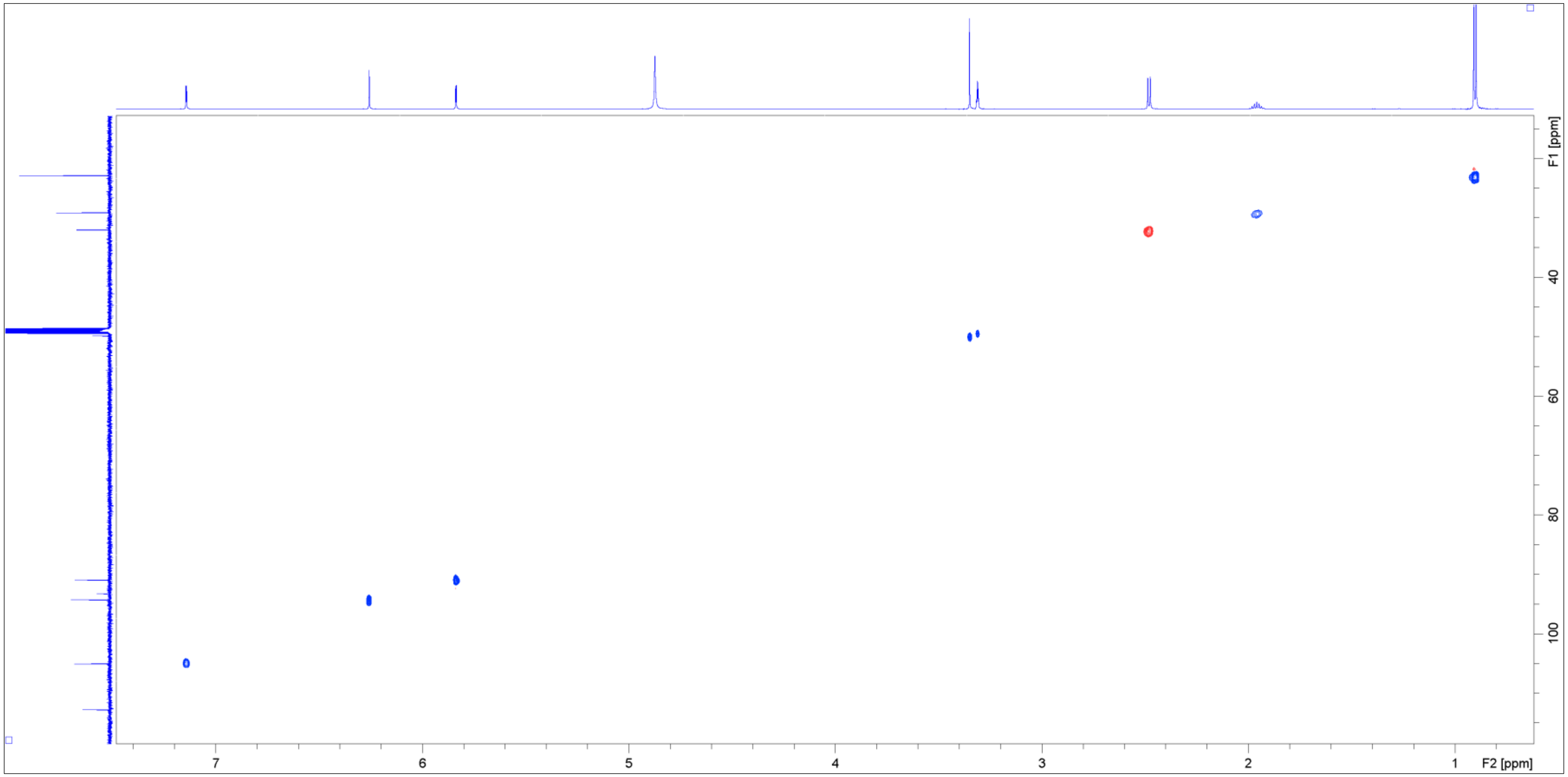

Figure S7. HSQC spectrum of **1** (CD_3OD).

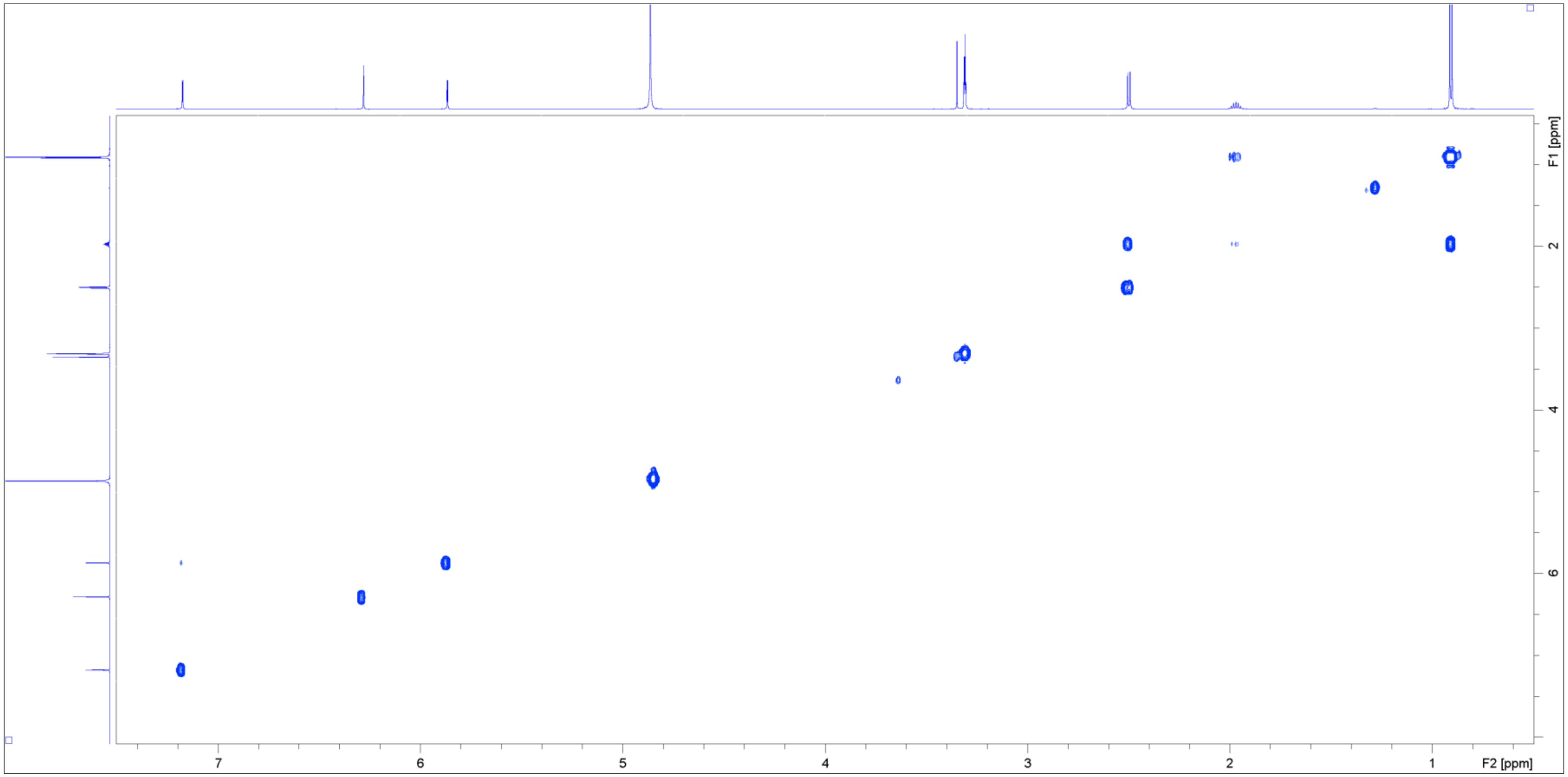

Figure S8. ^{1}H-^{1}H COSY spectrum of **1** (CD_3OD).

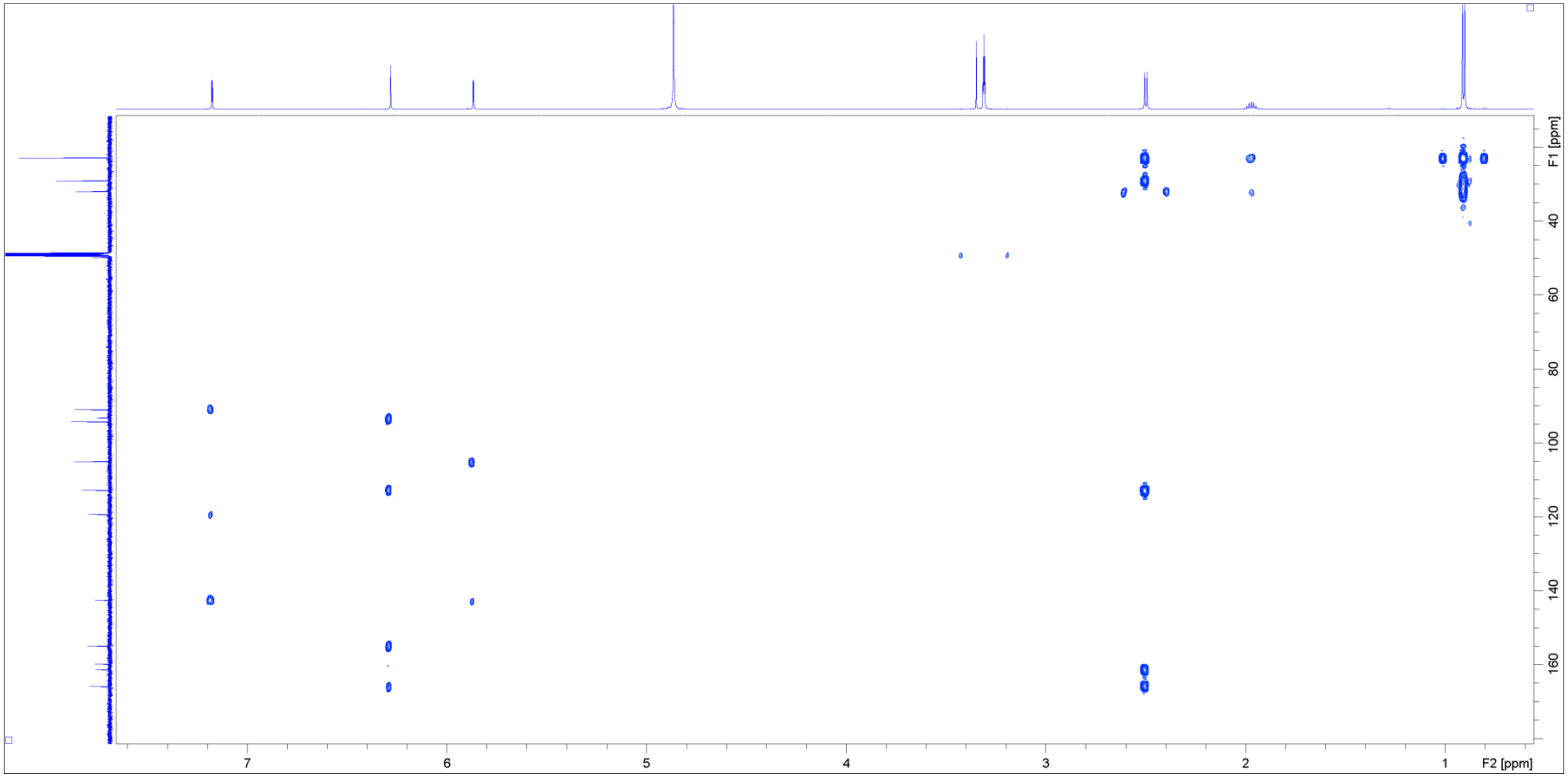

Figure S9. HMBC spectrum of **1** (CD_3OD).

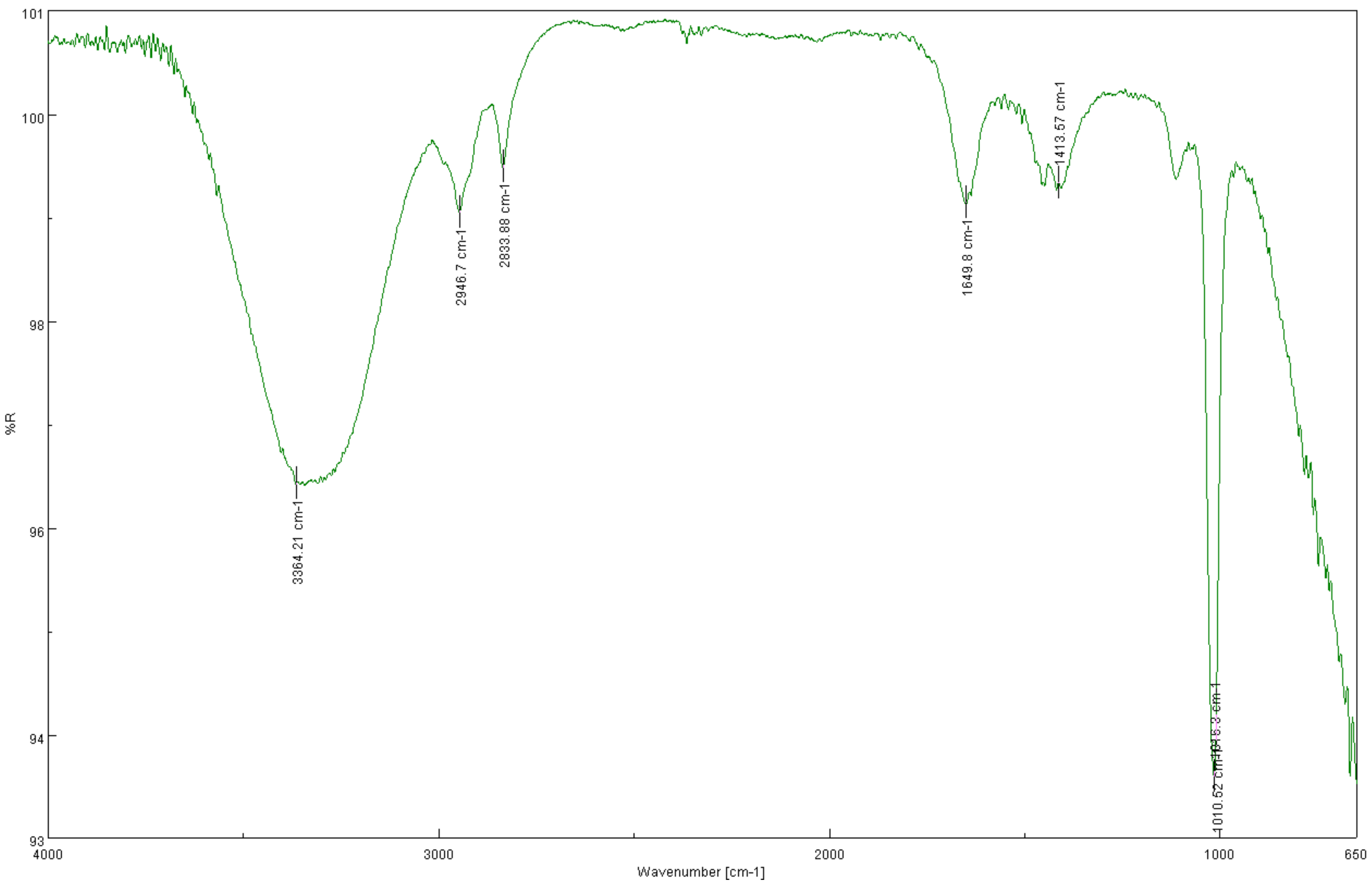

Figure S10. IR spectrum of **2**.

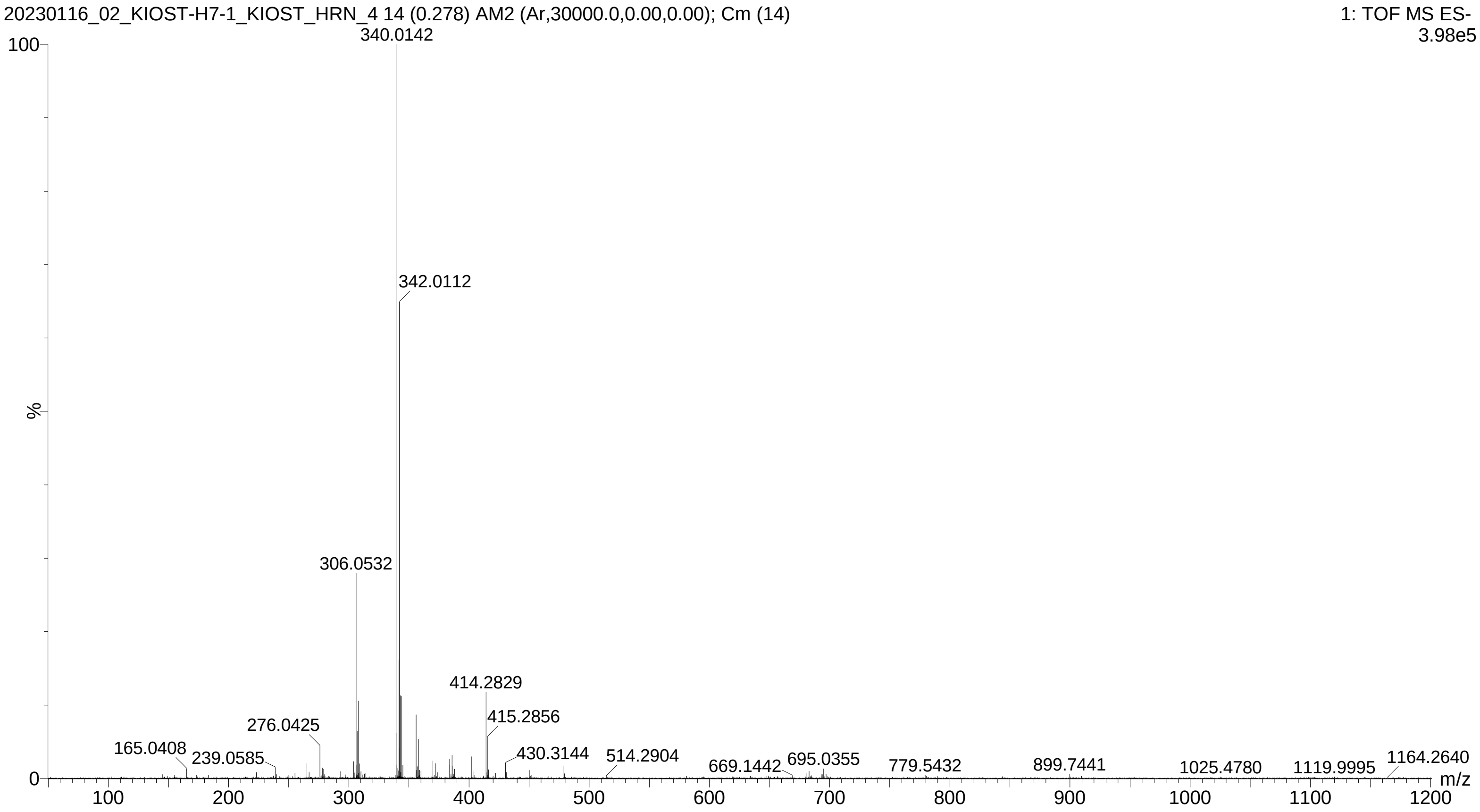

Elements Used:

C: 0-20 H: 0-180 N: 0-2 O: 0-5 Cl: 0-2

Minimum:				-9.0				
Maximum:		5.0	5.0	100.0				
Mass	Calc. Mass	mDa	PPM	DBE	i-FIT	Norm	Conf(%)	Formula
340.0142	340.0143	-0.1	-0.3	9.5	991.2	n/a	n/a	C15 H12 N O4 Cl2

Figure S11. HRESIMS data of **2**.

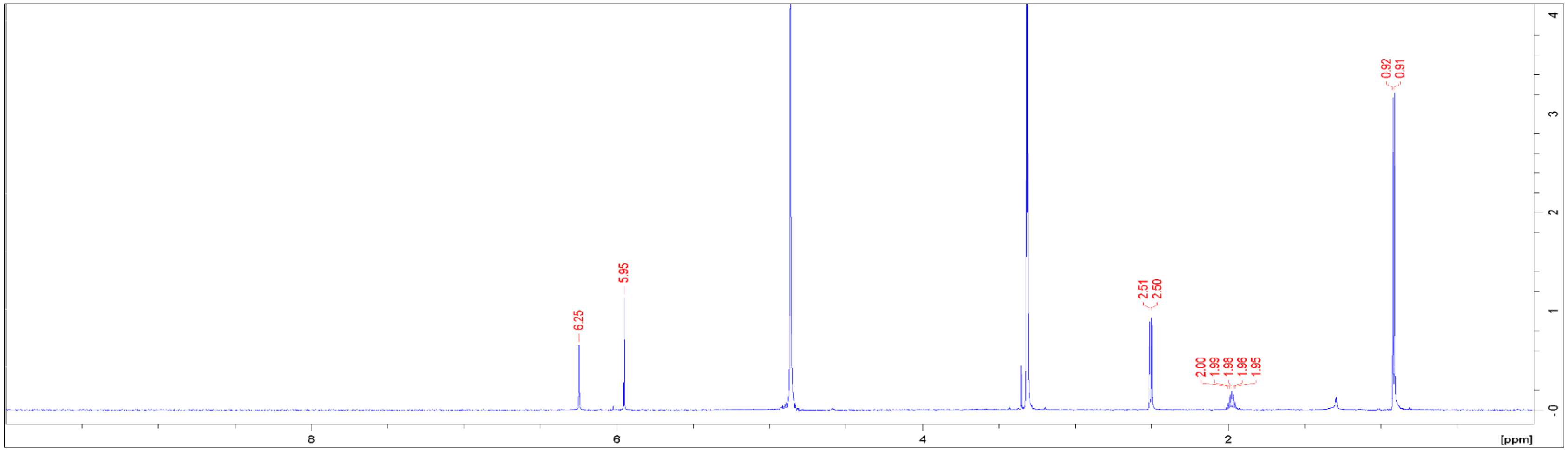

Figure S12. ^{1}H NMR spectrum of **2** (CD_3OD).

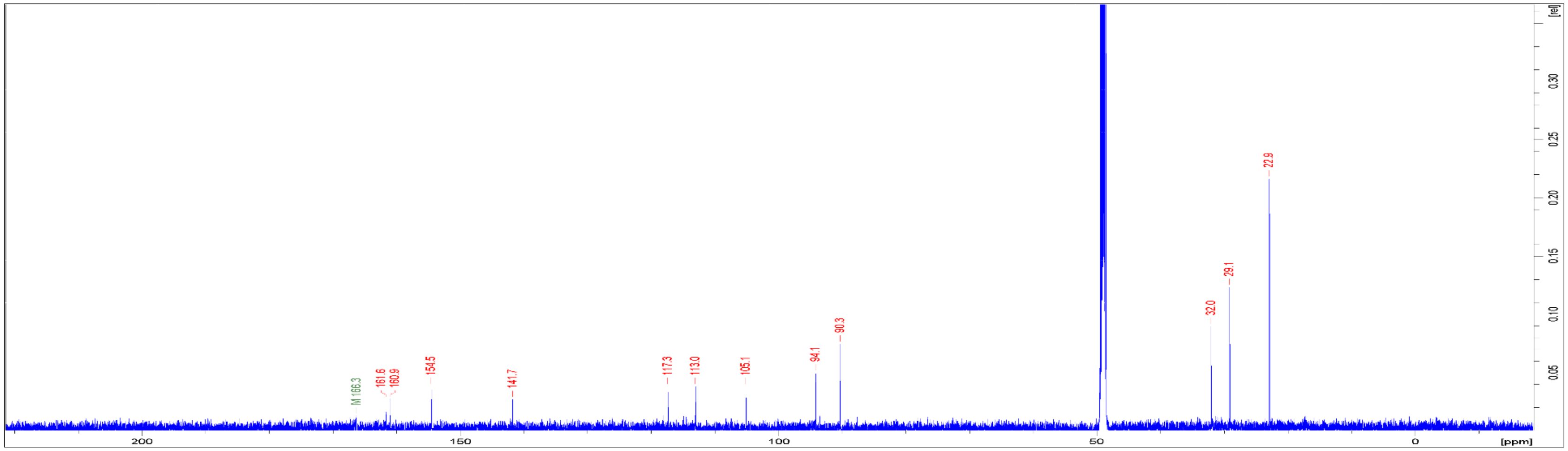

Figure S13. ^{13}C NMR spectrum of **2** (CD_3OD).

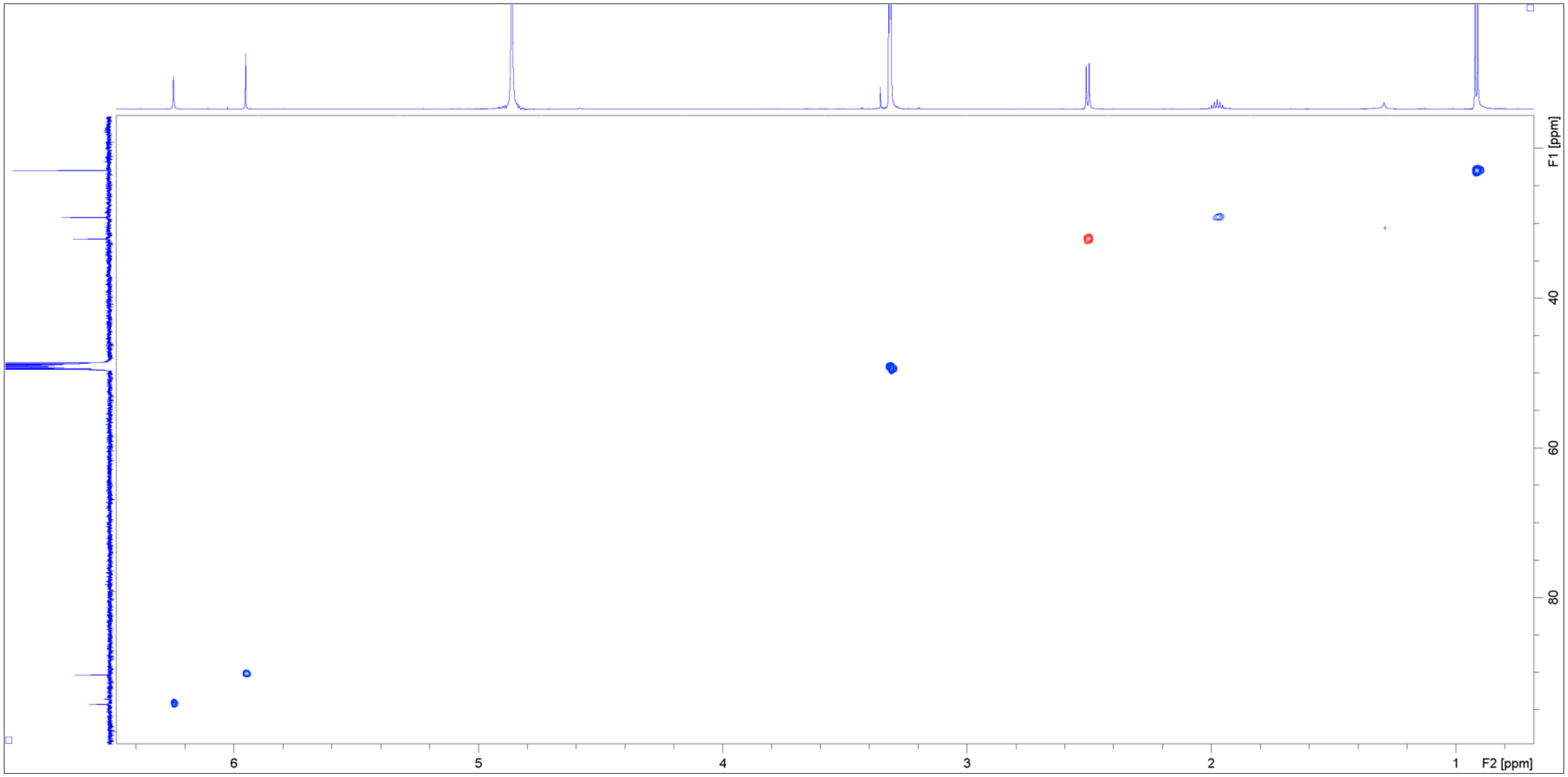

Figure S14. HSQC spectrum of **2** (CD_3OD).

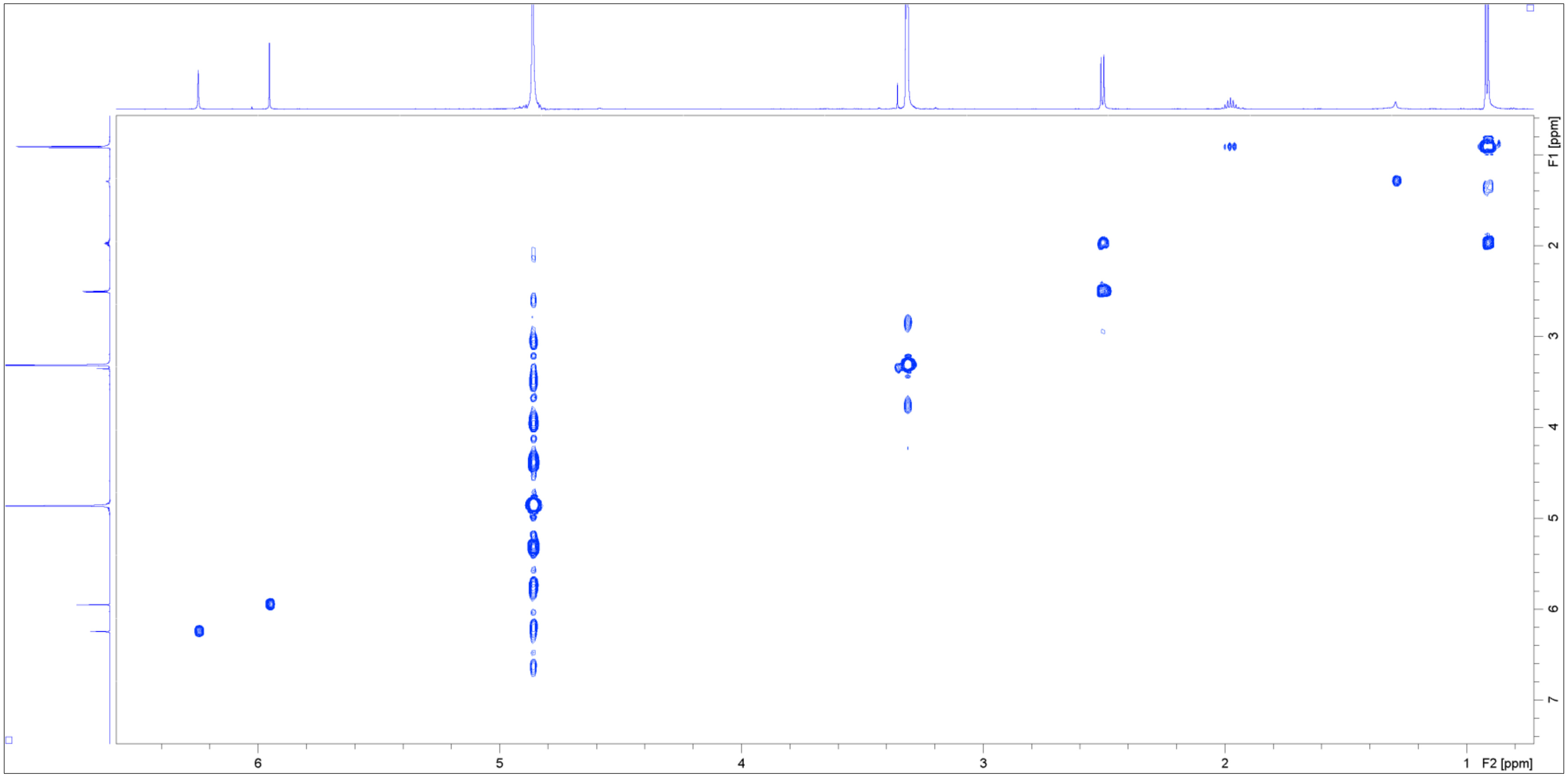

Figure S15. ^{1}H-^{1}H COSY spectrum of **2** (CD_3OD).

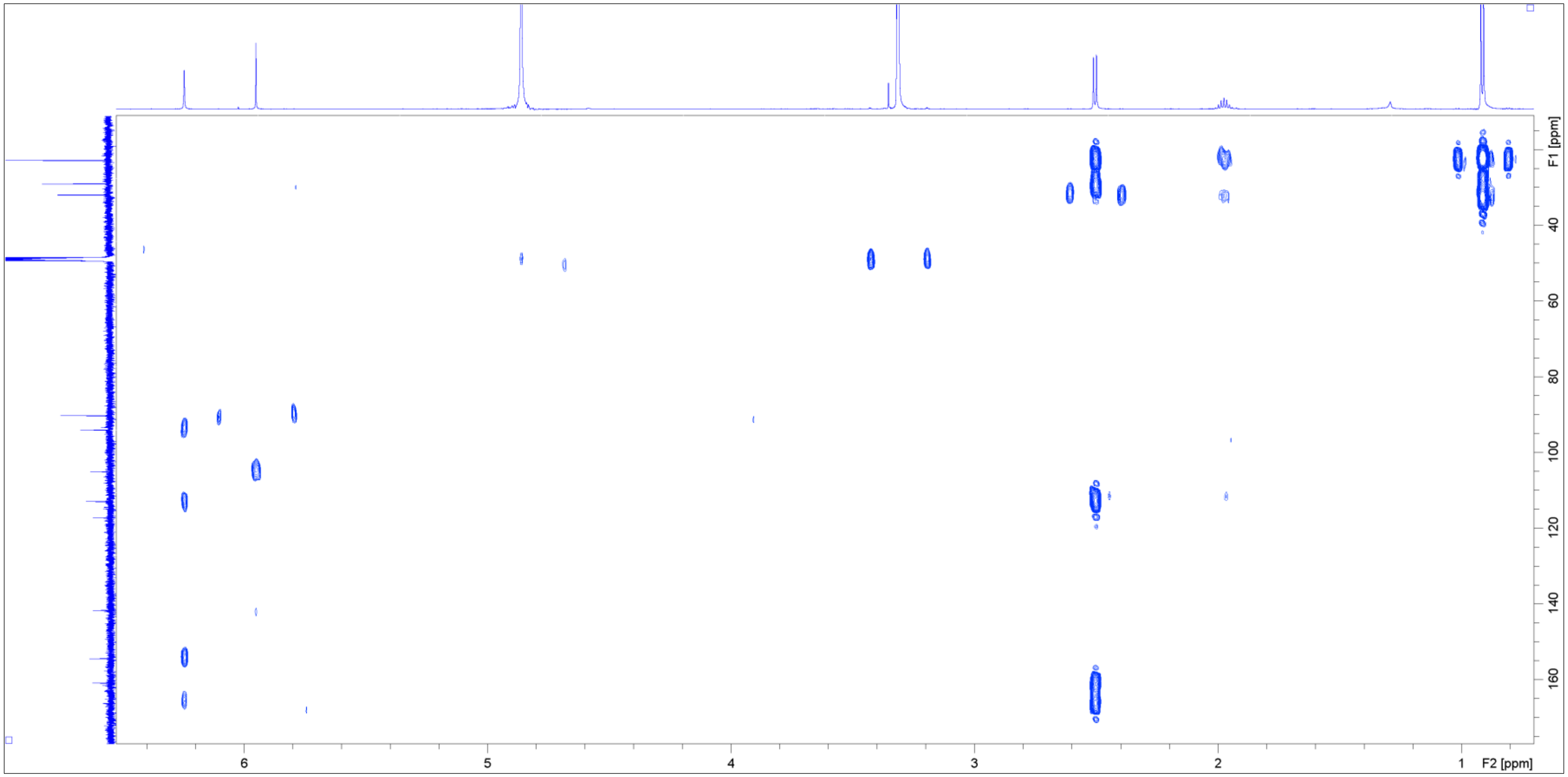

Figure S16. HMBC spectrum of **2** (CD_3OD).

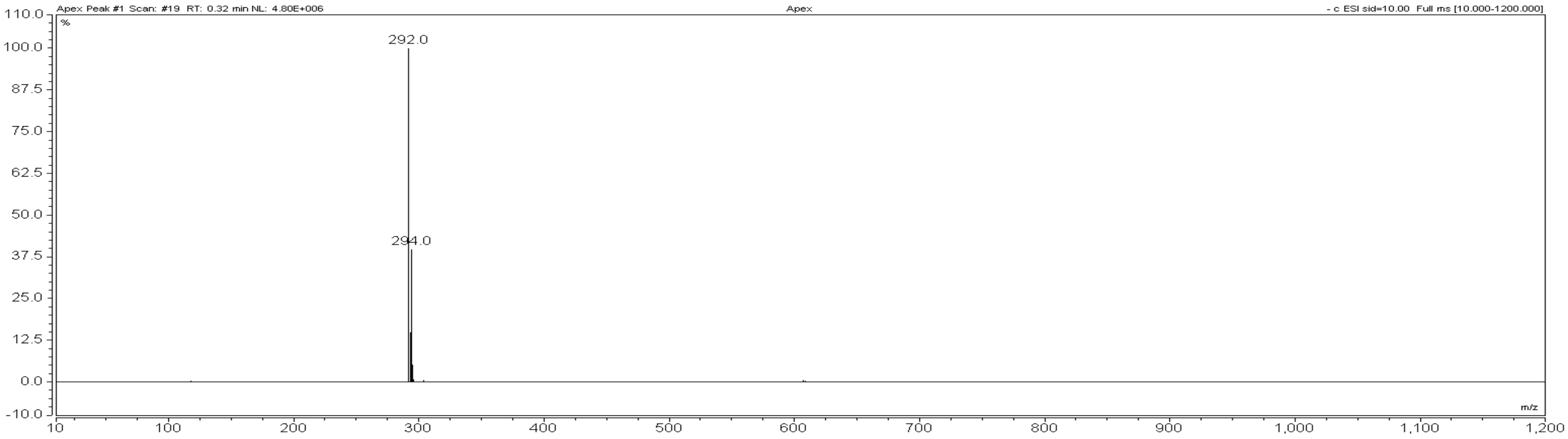

Figure S17. LRESIMS data of **3**.

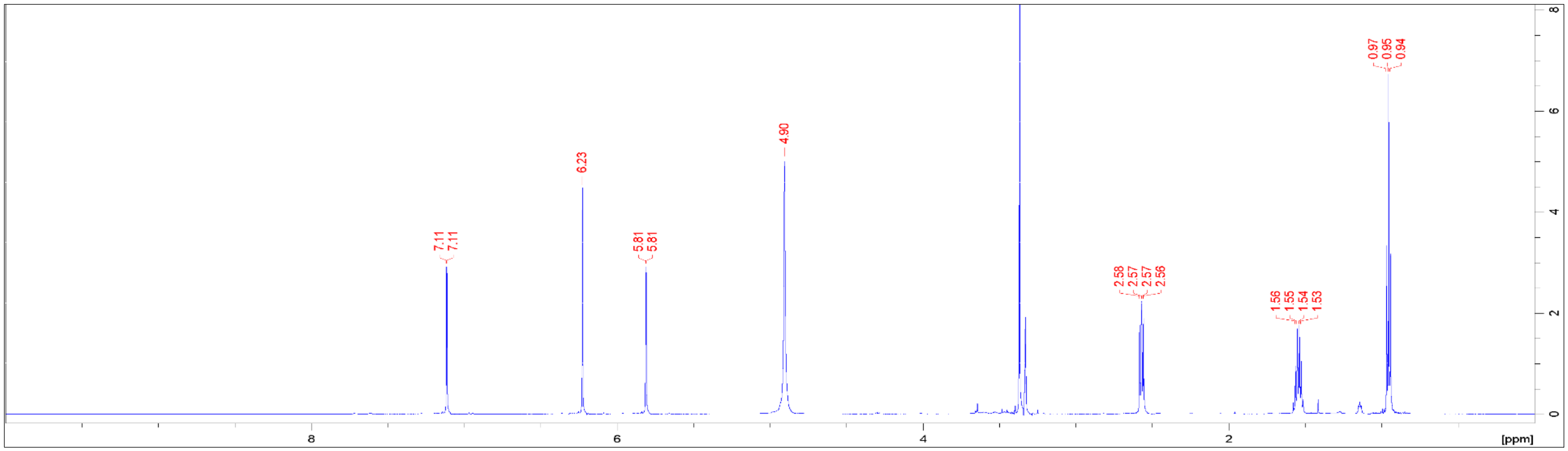

Figure S18. ^{1}H NMR spectrum of **3** (CD_3OD).

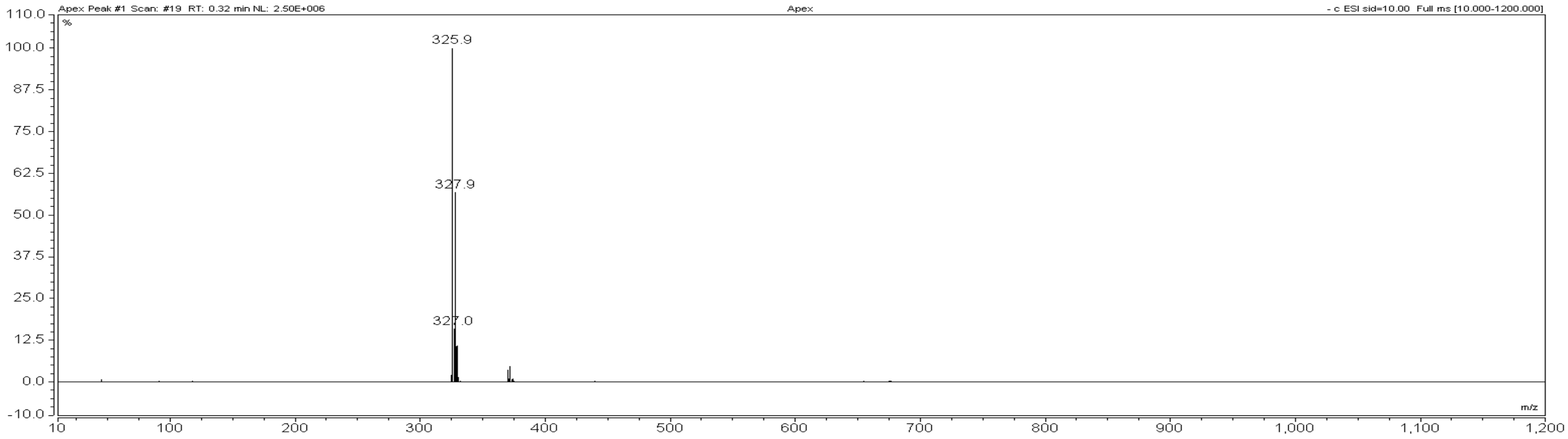

Figure S19. LRESIMS data of **4**.

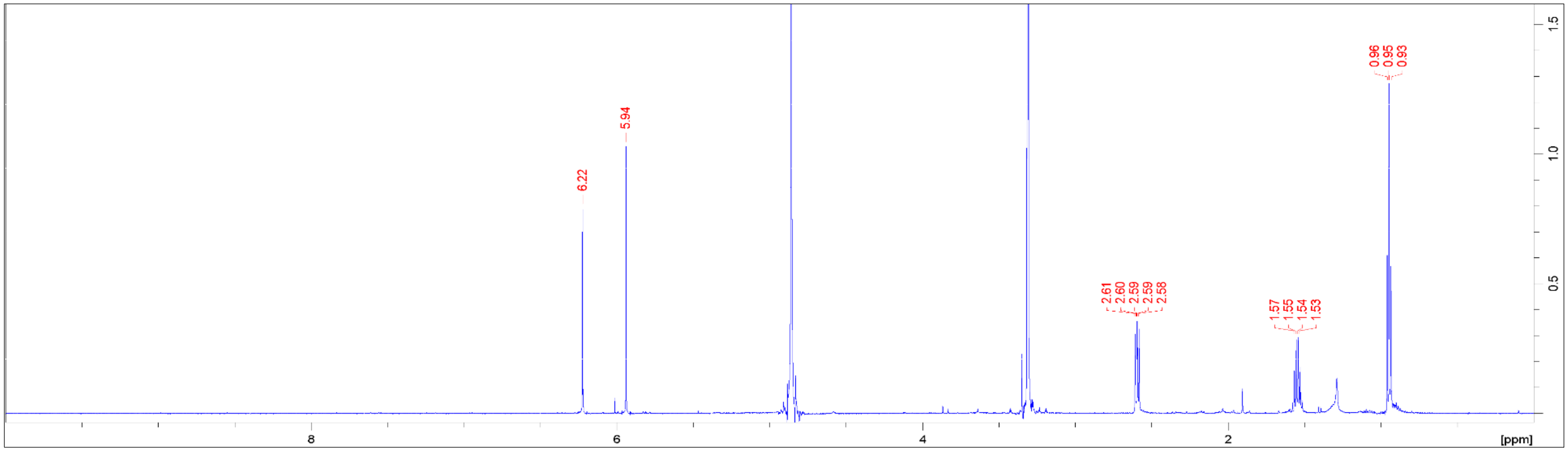

Figure S20. ^{1}H NMR spectrum of **4** (CD_3OD).

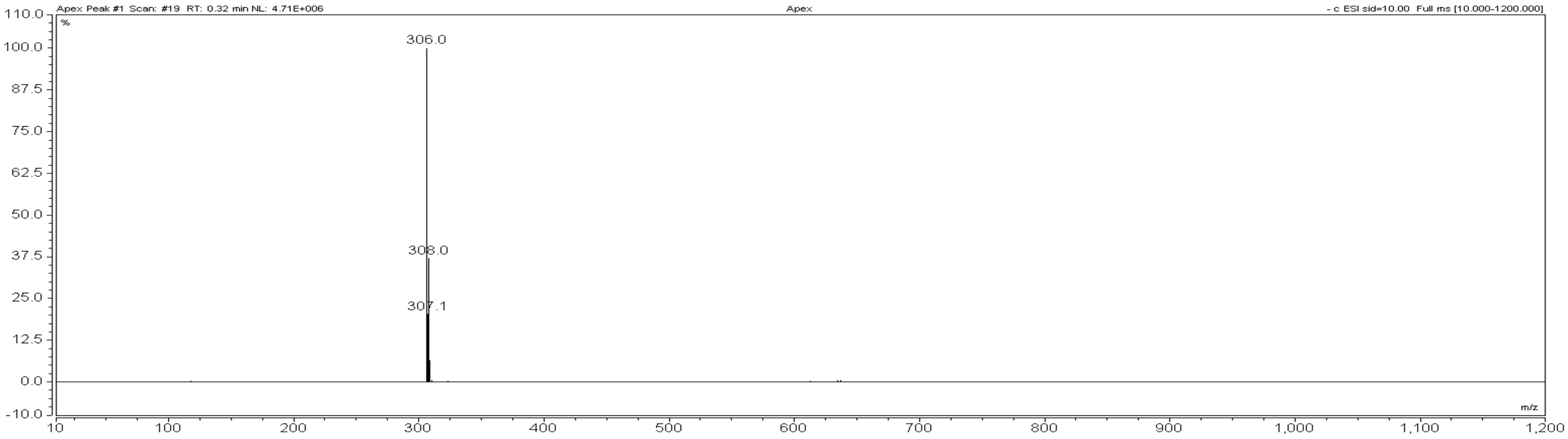

Figure S21. LRESIMS data of **5**.

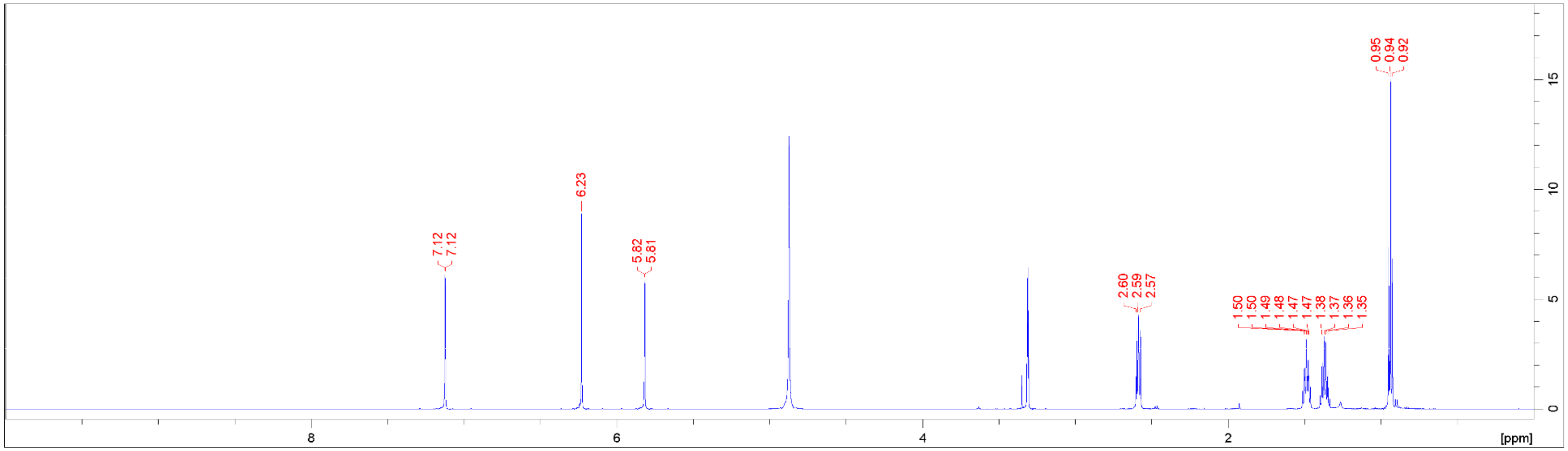

Figure S22. ^{1}H NMR spectrum of **5** (CD_3OD).

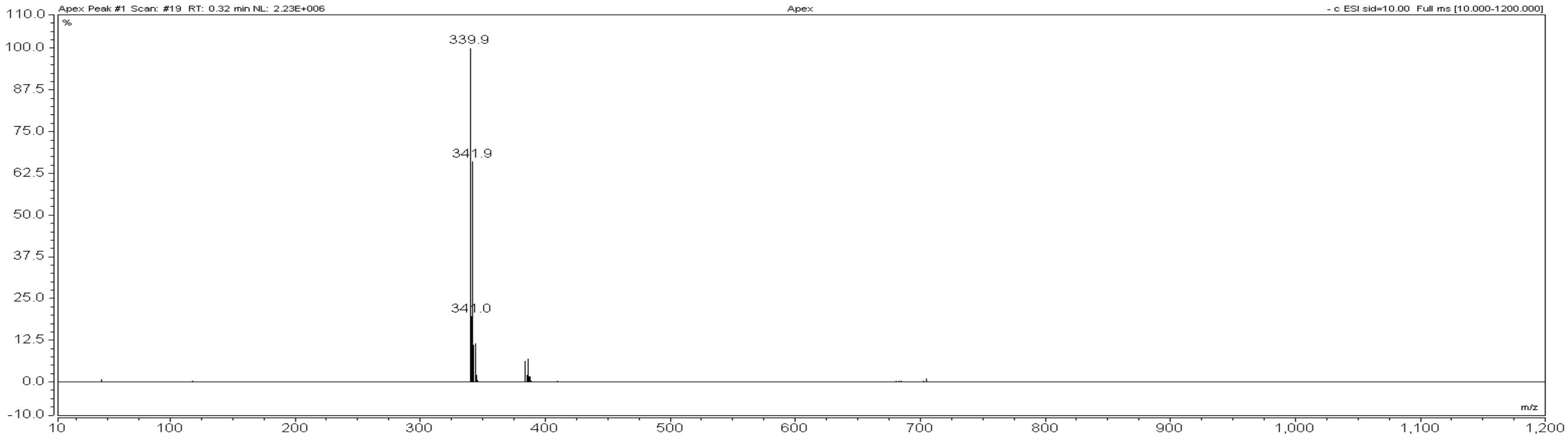

Figure S23. LRESIMS data of **6**.

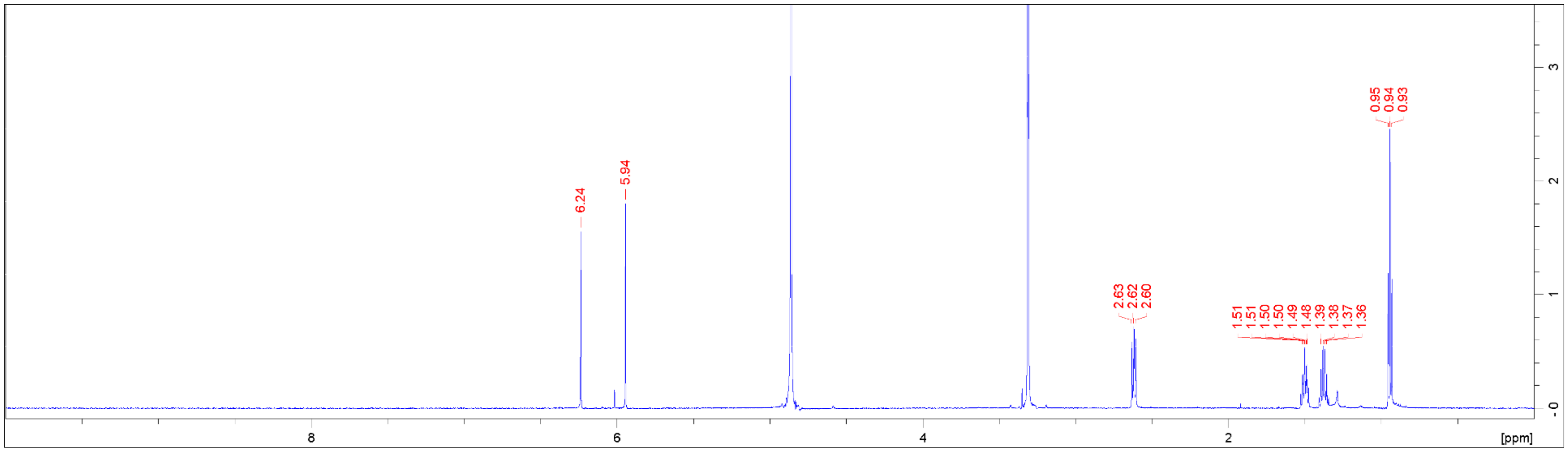

Figure S24. ^{1}H NMR spectrum of **6** (CD_3OD).

		1		3		5			Adriamycin	
	Conc.(ug/ml)	Mean	SD	Mean	SD	Mean	SD	Conc.(ug/ml)	Mean	SD
	30	-28.74	5.55	-29.22	4.16	-30.03	4.21	3	-7.74	5.15
	10	-4.22	2.35	0.21	7.58	-9.07	5.72	1	-5.83	3.55
ACHN	3	21.67	7.28	19.01	4.65	61.02	7.53	0.3	25.55	3.87
	1	68.73	7.02	82.14	6.25	87.32	3.88	0.1	37.90	3.82
	0.3	93.82	1.63	96.65	4.13	99.22	4.66	0.03	96.14	4.93
	GI_{50}	1.490		1.751		3.222		GI_{50}	0.093	
	GI_{50} (μM)	4.853		5.976		10.495		GI_{50} (μM)	0.171	

		1		3		5			Adriamycin	
	Conc.(ug/ml)	Mean	SD	Mean	SD	Mean	SD	Conc.(ug/ml)	Mean	SD
	30	-29.49	4.55	-31.21	1.43	-30.78	5.54	3	-10.35	3.72
	10	-4.42	8.20	0.22	6.90	-10.99	4.83	1	-3.01	3.69
MDA-MB-231	3	29.00	2.54	38.85	6.95	66.67	4.04	0.3	17.16	9.01
	1	83.30	5.26	76.13	10.41	85.46	4.33	0.1	28.58	8.16
	0.3	95.06	6.10	93.22	6.77	97.80	1.24	0.03	95.99	4.65
	GI_{50}	2.000		2.080		3.290		GI_{50}	0.075	
	GI_{50} (μM)	6.514		7.099		10.717		GI_{50} (μM)	0.138	

		1		3		5			Adriamycin	
	Conc.(ug/ml)	Mean	SD	Mean	SD	Mean	SD	Conc.(ug/ml)	Mean	SD
	30	-31.83	3.48	-28.51	4.28	-30.61	4.62	3	-9.55	1.82
	10	-2.76	2.21	1.84	9.77	-6.32	1.20	1	-4.42	4.11
PC-3	3	26.11	6.74	33.93	3.44	60.15	6.97	0.3	19.99	8.62
	1	82.66	1.89	86.48	2.56	91.92	9.13	0.1	32.49	5.50
	0.3	97.38	3.62	99.98	2.70	98.51	3.37	0.03	95.70	4.63
	GI_{50}	1.920		2.244		3.207		GI_{50}	0.080	
	GI_{50} (μM)	6.253		7.659		10.446		GI_{50} (μM)	0.147	

		1		3		5			Adriamycin	
	Conc.(ug/ml)	Mean	SD	Mean	SD	Mean	SD	Conc.(ug/ml)	Mean	SD
	30	-30.32	5.20	-33.45	6.28	-38.09	4.87	3	-11.43	5.75
	10	0.89	8.76	-5.92	6.87	-13.38	6.39	1	-4.75	6.54
NUGC-3	3	19.21	5.82	27.13	4.36	57.31	4.16	0.3	17.63	6.64
	1	76.37	6.79	80.05	5.94	80.91	8.04	0.1	34.07	7.02
	0.3	97.61	0.71	97.74	4.02	96.33	4.64	0.03	97.35	2.44
	GI_{50}	1.630		1.877		3.189		GI_{50}	0.082	
	GI_{50} (μM)	5.309		6.406		10.388		GI_{50} (μM)	0.151	

		1		3		5			Adriamycin	
	Conc.(ug/ml)	Mean	SD	Mean	SD	Mean	SD	Conc.(ug/ml)	Mean	SD
	30	-28.94	7.58	-24.36	6.08	-34.25	5.35	3	-14.08	3.85
	10	-3.66	3.18	-8.39	3.72	-8.72	3.68	1	-3.76	3.44
NCI-H23	3	22.53	7.88	21.86	5.63	63.95	4.15	0.3	17.16	2.00
	1	83.98	4.64	89.50	9.52	84.30	5.92	0.1	25.42	4.80
	0.3	97.69	6.02	95.48	5.90	97.62	3.90	0.03	96.66	6.95
	GI_{50}	1.870		1.989		3.306		GI_{50}	0.073	
	GI_{50} (μM)	6.090		6.788		10.769		GI_{50} (μM)	0.133	

		1		3		5			Adriamycin	
	Conc.(ug/ml)	Mean	SD	Mean	SD	Mean	SD	Conc.(ug/ml)	Mean	SD
	30	-20.09	5.80	-24.34	5.12	-31.36	9.41	3	-13.83	4.65
	10	-5.12	9.49	-0.66	5.47	-7.96	7.10	1	-8.68	4.34
HCT-15	3	26.21	4.51	30.04	2.46	65.71	4.08	0.3	19.48	8.19
	1	87.29	4.72	92.17	6.01	84.54	8.37	0.1	38.73	2.13
	0.3	100.05	3.75	99.55	3.86	98.21	3.63	0.03	97.25	4.40
	GI_{50}	2.040		2.270		3.290		GI_{50}	0.089	
	GI_{50} (μM)	6.644		7.747		10.717		GI_{50} (μM)	0.164	

Table S1. Results of the cytotoxicity test of **1**,**3** and **5**.
